# Impact of Elexacaftor/Tezacaftor/Ivacaftor Therapy on the Cystic Fibrosis Airway Microbial Metagenome

**DOI:** 10.1128/spectrum.01454-22

**Published:** 2022-09-26

**Authors:** Sophia T. Pallenberg, Marie-Madlen Pust, Ilona Rosenboom, Gesine Hansen, Lutz Wiehlmann, Anna-Maria Dittrich, Burkhard Tümmler

**Affiliations:** a Department for Pediatric Pneumology, Allergology and Neonatology, Hannover Medical School, Hannover, Germany; b German Center for Lung Research, Biomedical Research in Endstage and Obstructive Lung Disease (BREATH), Hannover Medical School, Hannover, Germany; c Research Core Unit Genomics, Hannover Medical School, Hannover, Germany; Emory University School of Medicine

**Keywords:** CFTR modulation, ELX/TEZ/IVA, airway metagenome, cystic fibrosis, metagenomics, whole-genome sequencing

## Abstract

The introduction of mutation-specific combination therapy with the cystic fibrosis transmembrane conductance regulator (CFTR) modulators elexacaftor/tezacaftor/ivacaftor (ELX/TEZ/IVA) has substantially improved lung function and quality of life of people with cystic fibrosis (CF). Collecting deep cough swabs and induced sputum, this postapproval study examined the effect of 14- and 50-week treatment with ELX/TEZ/IVA on the airway microbial metagenome of pancreatic- insufficient CF patients aged 12 years and older. Compared to pretreatment, the total bacterial load decreased, the individual species were more evenly distributed in the community, and the individual microbial metagenomes became more similar in their composition. However, the microbial network remained vulnerable to fragmentation. The initial shift of the CF metagenome was attributable to the ELX/TEZ/IVA-mediated gain of CFTR activity followed by a diversification driven by a group of commensals at the 1-year time point that are typical for healthy airways.

**IMPORTANCE** Shotgun metagenome sequencing of respiratory secretions with spike-in controls for normalization demonstrated that 1 year of high-efficient CFTR modulation with elexacaftor/tezacaftor/ivacaftor extensively reduced the bacterial load. Longer observation periods will be necessary to resolve whether the partial reversion of the basic defect that is achieved with ELX/TEZ/IVA is sufficient in the long run to render the CF lungs robust against the recolonization with common opportunistic pathogens.

## INTRODUCTION

Cystic fibrosis (CF) is a severe ion channel disease of autosomal recessive inheritance that is caused by mutations in the cystic fibrosis transmembrane conductance regulator (*CFTR*) gene (1, 2). Thanks to continuously improved symptomatic treatment during the last five decades, this lethal pediatric disease has been transformed into a chronic disorder with a median life expectancy of more than 50 years (1).

Chronic airway infections with opportunistic bacterial pathogens sustain a vicious cycle of infection, inflammation, and tissue damage and constitute the major comorbidity that limits the prognosis and quality of life of most people with CF (2). The CF lung microbiome typically consists of polymicrobial communities with dozens of bacterial species, viruses, and fungi (3–10). Whereas the taxonomic composition of the bacterial communities in CF airways has mainly been studied by 16S ribosomal DNA amplicon sequencing (5–8, 10–27), whole-genome shotgun sequencing (WGS) is taxonomically agnostic and captures viruses, bacteria, and fungi (28). The focus of past CF microbial airway metagenome studies has been on small cohorts of CF adolescents and adults who naturally expectorate comparably large volumes of respiratory secretions (29–39). We have optimized our wet-lab and *in silico* protocols so that the taxonomic and functional identification of core and rare species from shotgun metagenome sequencing data has become feasible with samples retrieved from all age groups (40–42).

In the past decades, therapy of CF was only symptomatic, but meanwhile, integration of mutation-specific CFTR modulators into clinical practice has achieved treatment of the basic defect of impaired epithelial conductance for chloride and bicarbonate in the majority of all CF patients (1, 2, 43). CFTR-potentiators increase the activity of CFTR at the cell surface, while CFTR-correctors facilitate the translation, folding, maturation, and trafficking of mutant CFTR to the cell surface and/or prevent its premature degradation (44). Ten years ago, the potentiator ivacaftor (IVA), the first mutation-type specific medication, was been approved for the treatment of patients carrying a gating mutation such as p.Gly551Asp or p.Arg117His in at least one of their two *CFTR* alleles (45–48). Meanwhile, the triple combination of the ivacaftor and the two correctors elexacaftor (ELX) and tezacaftor (TEZ) is available for the treatment of the more than 90% of people with CF (49–52).

Treatment of CF carriers of the gating mutation p.Gy551Asp with ivacaftor normalized the basic defect of chloride reabsorption in the sweat gland and attenuated multiple clinical symptoms of intestinal, upper, and lower airway disease (44). However, the effect on the CF lung microbiome was only modest. One-to 12-month treatment with ivacaftor did not modulate the airway microbiome (53–55), although long-term treatment reduced the prevalence of typical pathogens such as S. aureus, P. aeruginosa, and Aspergillus spp. in culture-based diagnostics of respiratory secretions (56). Upon initiation of ivacaftor therapy, the bacterial load with P. aeruginosa in chronically infected subjects was rapidly reduced (57), but P. aeruginosa density in sputum rebounded after 1 year, and P. aeruginosa clones that were present at pretreatment were found to persist for 6 years of follow-up (58). Hence, more recently, the hypothesis was tested whether ivacaftor combined with a 3.5-month intensive antibiotic course could clear chronic airway infections of individuals with CF carrying one or two p.Arg117His *CFTR* alleles (59). Ten of the 12 study participants remained P. aeruginosa positive or S. aureus positive for 2.5 years of follow-up. Interestingly, the two strongest responders to the ivacaftor-mediated modulation of the basic defect persistently cleared the pathogens from their airways.

The triple therapy with elexacaftor/tezacaftor/ivacaftor (ELX/TEZ/IVA) has been shown to be similarly efficacious for the large group of patients with one or two p.Phe508del alleles (49–52) as the monotherapy with ivacaftor for eligible patients (45–47). The immediate strong improvements in anthropometry, lung function, and sweat chloride seen upon initiation of triple therapy have inspired CF physicians to classify the triple combination as highly efficient modulator therapy (HEMT) (60, 61). Hence, we embarked on a postapproval study on whether HEMT was able to transform the CF airway metagenome. By applying WGS to respiratory secretions, we examined CF patients eligible for ELX/TEZ/IVA therapy pre-HEMT and after 3 to 4 and 11 to 13 months of HEMT in the composition of their microbial airway communities. In parallel, the study participants were assessed for lung function and CFTR biomarkers (62) so that any changes in the airway metagenome could be compared with the impact of HEMT on lung disease and the basic defect. In general, we observed a remarkable shift toward a healthy airway microbiome driven by the HEMT-mediated partial reversion of the basic defect, but the network of commensals remained vulnerable to fragmentation.

## RESULTS

### Characteristics of the study population and clinical outcome.

**FIG 1:**
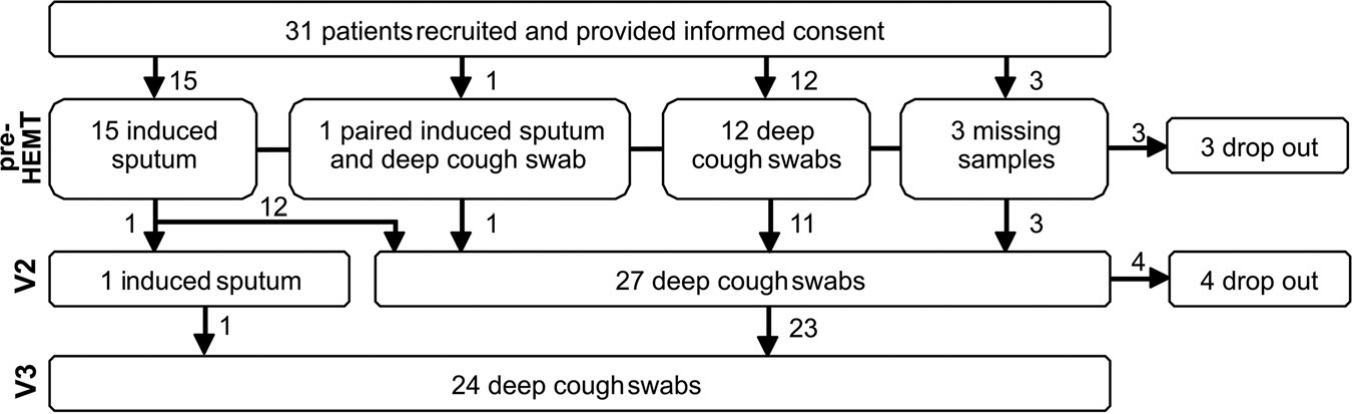
Flow chart of recruited CF study participants and obtained samples.

We enrolled 31 exocrine pancreatic insufficient people with CF (PI CF), including 18 women and 13 men (Fig. 1 and Table 1). Thirteen patients were homozygous for p.Phe508del (F/F), and 18 patients were compound heterozygous for p.Phe508del and a minimal function mutation (F/MF). The age range was 12.1 to 44.8 years, with a median age of 16.2 years.

**TABLE 1:** Clinical, functional, and microbial parameters of CF patients at V1, V2, and V3^*a*^

Study parameters	V1 [median (range) or *n* (%)]	V2 [median (range) or *n* (%)]	V3 [median (range) or *n* (%)]
No. of patients (*n*)	31	30	24
Age (yr)	16.2 (12.1 to 44.8)		
Sex (female)	18 (58%)	17 (57%)	15 (63%)
Pancreatic insufficiency (%)	31 (100%)	30 (100%)	24 (100%)
LUM/IVA therapy (*n*)	6 (19%)	0	0
TEZ/IVA therapy (*n*)	2 (6%)	0	0
Clinical parameters			
Leeds criteria for chronic P. aeruginosa colonization (chronic/intermittend/free/never)	11/2/13/5		
BMI (kg/m²)	19.8 (14.2 to 28.1)	20.1 (16.1 to 29.5)	20. 7 (16.1 to 26.5)
ΔBMI (kg/m²) to pre-HEMT		+0.7 (−1.2 to 3.5)	+0.6 (−1.2 to 4.2)
FEV_1_ (%)	85 (46 to 129)	106 (62 to 147)	105 (60 to 143)
ΔFEV_1_ (%) to pre-HEMT		+16 (0 to 36)	+16 (−11 to 44)
MEF_25_ (%)	39 (13 to 99)	91 (22 to 211)	100 (1 to 187)
ΔMEF_25_ (%) to pre-HEMT		+29 (−25 to 140)	+26 (−4 to 116)
Sweat chloride (mmol/L)	100 (75 to 115)	45.5 (10 to 101)	
ΔSweat chloride (mmol/L) to pre-HEMT		−53 (−8 to −89)	
Sermet score	−0.78 (−2.00 to 0.60)	0.18 (−1.09 to 1.58)	
ΔSermet score to pre-HEMT		+1.07 (−0.96 to +2.24)	
Metagenome diversity parameters (all samples)			
Species richness (*n*)	39 (7 to 137)	33 (0 to 124)	33 (7 to 127)
Pielou’s evenness	0.64 (0.02 to 0.96)	0.70 (0 to 0.91)	0.82 (0.50 to 0.93)
Shannon diversity index	2.24 (0.05 to 3.25)	2.62 (0 to 3.35)	2.73 (1.38 to 3.23)
Simpson diversity index	0.83 (0.01 to 0.95)	0.89 (0 to 1.00)	0.91 (0.63 to 0.95)
Bacterial DNA load (all samples)			
Total	52.44 (1.25 to 12,300)	19.61 (0 to 135)	1.71 (0.01 to 54.14)
P. aeruginosa (in pre-HEMT-positive patients)	31.96 (0.89 to 281.5)	0.004 (0 to 0.59)	0 (0 to 0.08)
S. aureus (in pre-HEMT-positive patients)	2.19 (0.01 to 42.78)	0 (0 to 0.05)	0 (0 to 0)
H. influenzae (in pre-HEMT-positive patients)	0.43 (0.01 to 141.5)	0.02 (0 to 0.95)	0.003 (0 to 0.36)

*^a^*LUM/IVA, lumacaftor/ivacaftor; TEZ/IVA, tezacaftor/ivacaftor; BMI, body mass index; FEV_1_, forced expiratory volume in 1 s capacity in percent predicted; MEF_25_, midexpiratory flow at 25% of vital capacity in percent predicted.

Pre-HEMT, eight F/F patients had already been on combination therapy with the CFTR modulators lumacaftor/ivacaftor (LUM/IVA; *n* = 6) (63) or tezacaftor/ivacaftor (TEZ/IVA; *n* = 2) (63). According to the clinical “Leeds” criteria (64), we enrolled 11 patients chronically colonized with P. aeruginosa (>50% positive cultures in the last 12 months). Intermittent colonization was present in two patients with less than 50% positive cultures in the last 12 months. Thirteen patients experienced previous colonization but had been free of P. aeruginosa for >12 months at study inclusion, while another five patients had no history of colonization (64) (Table 1). We obtained induced sputum in 16 patients, while in 12 patients, sputum induction failed, and thus deep cough swabs were collected (Fig. 1). All patients showed CFTR dysfunction pre-HEMT (V1) in the sweat gland according to their sweat chloride concentration and all but one in respiratory epithelia, as assessed by nasal transepithelial potential difference measurements (NPD) (44, 65–67) quantified by the Sermet Score (66) (Table 1; see also Table S1 in the supplemental material).

Clinical responses to ELX/TEZ/IVA (Table 1) after 14 ± 5 weeks of treatment (V2) showed a median increase in body mass index (BMI) of +0.7 kg/m^2^. Lung spirometry improved in terms of absolute percentage points by a median of 16% for median forced expiratory volume in 1 s (FEV_1_) and of 29% for midexpiratory flow at 25% (MEF_25_). Sweat chloride concentration decreased by a median of −53 mmol/l, while the CFTR function in the respiratory epithelium was improved by +1.1 in the Sermet Score (62) (Table 1). These improvements in anthropometry, lung function, and CFTR biomarkers were all highly significant (*P < *0.001, Wilcoxon signed-rank test). Sputum induction failed in all but one patient, leaving us to resort to deep cough swabs (*n* = 27, Fig. 1).

After 50 ± 14 weeks of ELX/TEZ/IVA therapy (V3), the median gains in body mass index (+0.6), FEV_1_ (+16%), and MEF_25_ (+26%) were maintained (Table 1). Of the 31 study participants, values in the normal range of FEV_1_ (>80%), MEF_25_ (>80%), sweat chloride (<30 mmol/l), and Sermet score (>0.27) were recorded during triple therapy in 28, 17, 7, and 12 patients, respectively (Table 1, Table S1). Deep cough swabs (*n* = 24) were collected in all patients due to failure of sputum induction (Fig. 1).

### Case reports.

**FIG 2:**
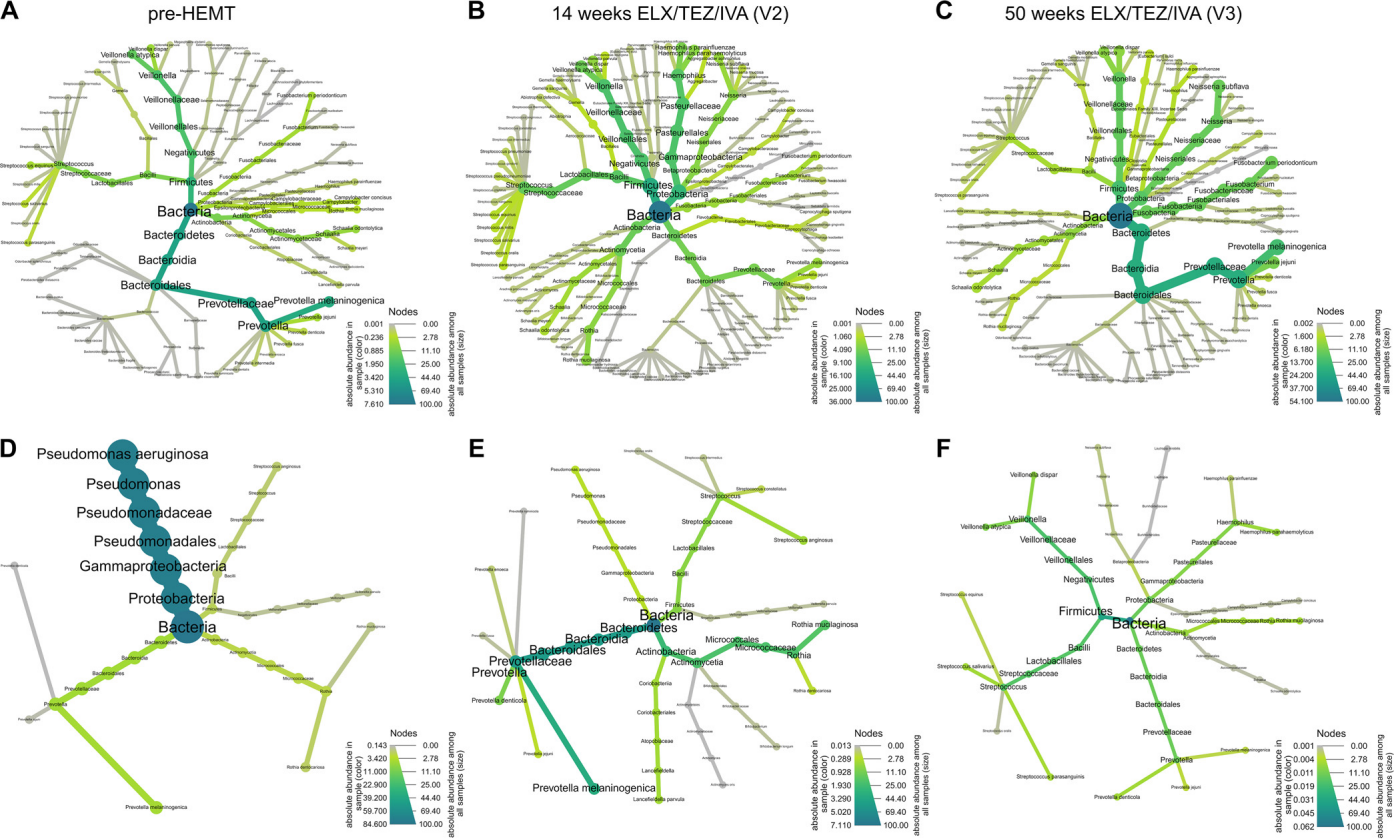
Heat tree representation (83, 84) of absolute species abundance in two highly discordant CF-patients (*CF-20*, top row; *CF-25*, bottom row) pre-HEMT (A and D), after 14 weeks (B and E), and after 50 weeks (C and F) of treatment with ELX/TEZ/IVA. The lung function of *CF-20* (12 years, F/MF, not pretreated with any CFTR modulator, no colonization with P. aeruginosa) was highly impacted by ELX/TEZ/IVA (FEV_1_, +29%; MEF_25_, +75%), whereas his airway metagenome diversified only slightly (Species numbers, +19; Pielou’s evenness, +0.19; Shannon diversity, +1.06; Simpson diversity, +0.15) (B and C). Conversely, in *CF-25* (44 years, F/F, pretreated with TEZ/IVA, chronically colonized with P. aeruginosa) with advanced CF lung disease and a low-diversity metagenome dominated by P. aeruginosa pre-HEMT (D), ELX/TEZ/IVA substantially diversified the airway metagenome (species numbers, +9; Pielou’s evenness, +0.70; Shannon diversity, +2.11; Simpson diversity, +0.73; total bacterial load, −84.5; P. aeruginosa bacterial load, −76.4 to 0) (E and F), whereas his lung function remained nearly unaffected (FEV_1_, +6%; MEF_25_, +3%). PCoA, principal coordinate analysis. Note: the species abundance values are based on sequin normalized data, i.e., the ratio of the number of reads assigned to the species to the number of the spiked-in internal reference standard (71). Sequins constitute a synthetic community of artificial microbial genomes that have been shown to accurately resolve the relative and absolute abundance of a taxon in a metagenome (71). Heat trees were generated by the Wochenende Pipeline (73). Absolute abundance in the single sample is coded by color, while absolute abundance among all samples is coded by size of the species name.

Before presenting the findings of our study, we would like to introduce the spectrum of individual responses to ELX/TEZ/IVA by comparing the results for the eldest study participant chronically colonized with P. aeruginosa (*CF-25*, age 44 years, F/F) with those for a P. aeruginosa-negative child (*CF-20*, age 12 years, F/MF). Under ELX/TEZ/IVA therapy, the child’s lung function improved into the normal range beyond 100% predicted, whereas his airway metagenome only slightly diversified (Fig. 2A to C) (Table S1 and S2).

Conversely, the 44-year-old male showed advanced CF lung disease with compromised spirometry and a low-diversity metagenome dominated by P. aeruginosa (Fig. 2D). One-year treatment with ELX/TEZ/IVA did not significantly affect the lung function (FEV_1_, +6%; MEF_25_, +3%), but substantially diversified the airway metagenome. Total bacterial load, mainly attributable to P. aeruginosa pre-HEMT, was significantly reduced, whereas typical commensals associated with a healthy microbiome (68, 69) were emerging (Fig. 2E and F). We learned from these discordant clinical phenotypes that on a case-to-case basis we cannot anticipate a predestined correlation between the patient’s individual responses to HEMT in lung function and airway microbial metagenome. However, as outlined in the next section, some general trends could be seen.

### Relative microbial abundances of core and rare species in CF airway metagenome.

**FIG 3:**
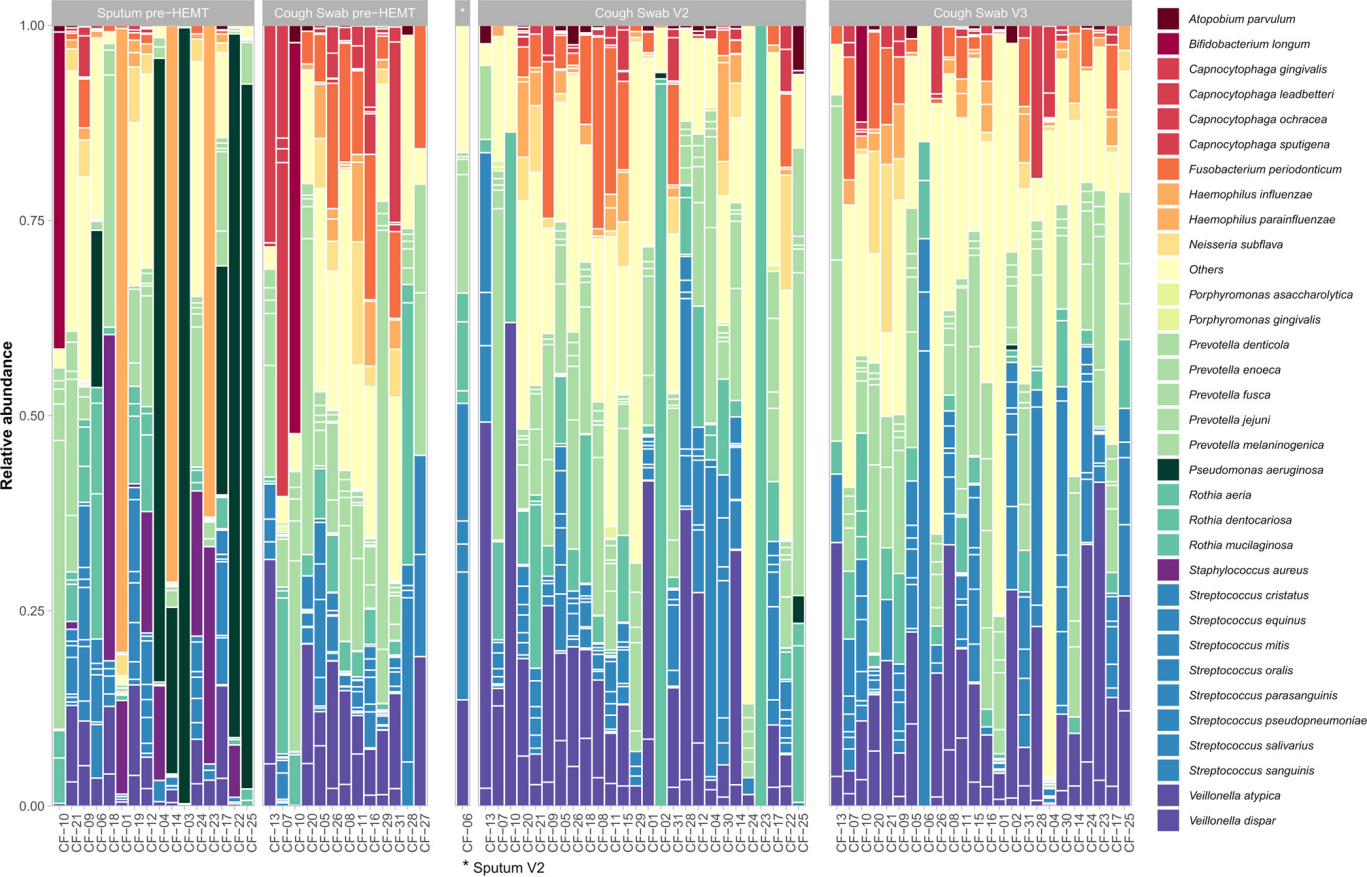
Overview of the relative microbial abundances of core and rare species in CF airway metagenome samples pre-HEMT, after 14 weeks (V2), and 50 weeks (V3) of treatment with ELX/TEZ/IVA. Note: relative abundances were calculated from sequins-normalized microbial count data. The bars and legend are sorted alphabetically. The colors represent taxonomic classification at the genus level. Samples are ordered by sample type, time point (V1, V2, and V3), and age and labeled with patients' pseudonyms. Rare species are defined as all species contributing to a total abundance of <5% in all samples and are summarized as “others.” The asterisk (*) marks the sputum sample at V2.

**FIG 4:**
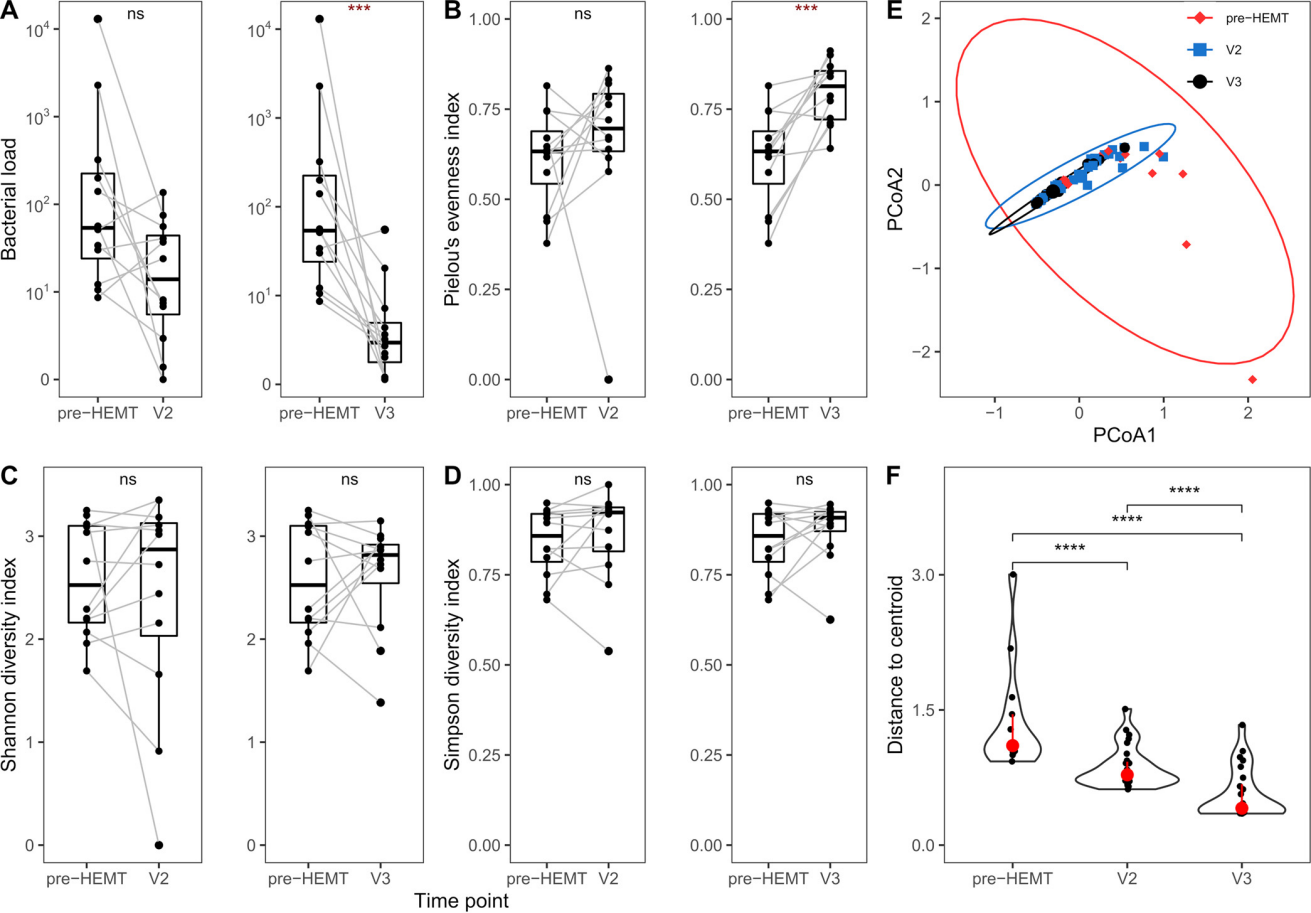
The effect of ELX/TEZ/IVA on alpha- and beta-diversity of the CF airway metagenome based on deep cough swabs. (A) Pairwise comparison of the total bacterial load between either pre-HEMT samples and samples collected at the first study visit (V2, Wilcoxon adjusted *P* value > 0.05) or between pre-HEMT samples and samples obtained at the second study visit (V3, Wilcoxon adjusted *P* value < 0.001; *r* = 0.74; confidence interval [CI] = 0.52–0.85) after commencement of ELX/TEZ/IVA treatment. (B) Pairwise comparison of Pielou’s evenness between pre-HEMT samples and samples obtained at V2 (Wilcoxon adjusted *P* value > 0.05) or V3 (Wilcoxon adjusted *P* value < 0.001; *r* = 0.66; CI = 0.36–0.83). (C) Pairwise comparison of Shannon diversity indices between either pre-HEMT samples or samples collected at the first study visit (V2, Wilcoxon adjusted *P* value > 0.05) or between baseline samples and samples obtained at the second study visit (V3, Wilcoxon adjusted *P* value > 0.05) after commencement of ELX/TEZ/IVA treatment. (D) Pairwise comparison of Simpsons diversity indices between pre-HEMT samples and samples obtained at V2 (Wilcoxon adjusted *P* value > 0.05) or V3 (Wilcoxon adjusted *P* value > 0.05). (E) Principal coordinate analysis based on a Euclidean-distance matrix obtained from log_10_-scaled absolute microbial abundance values per cough swab. (F) To test multivariate homogeneity of variances between pre-HEMT, V2, or V3 samples, the distance between each data point and its group centroid was obtained, and the data spread was compared between time points (Kruskal-Wallis, *P* value < 0.0001; epsilon-squared effect size = 0.49; CI = 0.31–0.68). ***, *P < *0.001; ****, *P < *0.0001.

Regarding relative abundances of core species pre-HEMT, we can differentiate between patients capable of producing sputum (*n* = 15) after induction, who were more likely to carry CF pathogens (P. aeruginosa, S. aureus, and H. influenzae), and patients who failed to produce sputum (*n* = 12, for detailed information on sampling see Fig. 1). Those patients providing deep cough swabs instead of sputum after inhalation with 3% NaCl (70) pre-HEMT showed species relative abundances more similar to the pattern observed after initiation of ELX/TEZ/IVA therapy (Fig. 3; for absolute abundances, see Fig. S1; for the statistical evaluation, see Fig. 4). The respiratory tract in 10 patients was dominated (relative abundance > 50% of total abundance) by a single species, either P. aeruginosa (*n* = 4), S. aureus (*n* = 1), H. influenzae (*n* = 3), Bifidobacterium longum (*n* = 1), *Capnocytophagia* spp. (*n* = 1), or *Prevotella* spp. (*n* = 1) (Fig. 3).

After 14 ± 5 weeks of ELX/TEZ/IVA, most patients failed to produce sputum, and the CF airway metagenome was therefore assessed almost exclusively by deep cough swabs after inhalation (*n* = 27/28). At V2, the airway metagenome predominantly consisted of commensals such as *Prevotella*, *Rothia*, Streptococcus, and *Veillonella* spp. (Fig. 3). The relative dominance of single species decreased overall (Fig. 3), where only two samples were dominated by Rothia mucilaginosa (*CF-02*) or showed a R. mucilaginosa monoculture (*CF-23*) at V2. The relative abundance of CF pathognomonic species P. aeruginosa and S. aureus decreased, resulting in lack of dominance in any patient.

After 1 year of ELX/TEZ/IVA, the relative abundance patterns for all patients became even more homogenous and balanced with a high proportion of commensals. None of the patients showed a dominance of single species (Fig. 3).

### Relative microbial abundances of rare species in CF airway metagenome.

In patients with highly dominant single species pre-HEMT, we saw only a minor contribution of the rare species (39) (Fig. 3) (median, 1.5%; range, 0 to 5%), which we defined as the less abundant species contributing to less than 5% of total abundance in all samples (Table S3). After 14 weeks of therapy with ELX/TEZ/IVA (median, 15%; range, 9 to 77%), and even more so after 50 weeks of treatment (median, 37%; range, 19 to 81%), compared to pre-HEMT, we observed a higher proportion of rare species in cough swabs collected from these 10 patients (Fig. 3).

### Impact of ELX/TEZ/IVA on the abundance of the common CF pathogens.

Modification of the abundance of pathogens substantially associated with CF lung disease is an essential outcome measure of the efficacy of CFTR modulator therapy (1, 59, 60). To specifically address the impact of ELX/TEZ/IVA treatment on the abundance of hallmark CF pathogens, we defined subcohorts as pathogen-positive or pathogen-negative subjects for subsequent analyses based on the detection of P. aeruginosa, S. aureus, and H. influenzae DNA pre-HEMT. Please note that the sample type for V1 (both sputum and deep cough swabs) and V2 and V3 (deep cough swabs) is not consistent in this analysis.

Seven patients showed evidence of P. aeruginosa in sequencing pre-HEMT. The load with a particular taxon was determined by the dimensionless ratio of the number of reads assigned to this taxon to the number of reads of the internal sequin reference standard (71). Therapy with ELX/TEZ/IVA resulted in a strong decrease in the median P. aeruginosa DNA load in these patients from 32 at V1 in sputum (*n* = 6) or deep cough swab (*n* = 1) to 0.004 (V2) and 0 (V3) in subsequent deep cough swabs, with 71% of patients showing no detectable P. aeruginosa DNA at V3. Similarly, for S. aureus (*n* = 11), the median load decreased from 2.2 at V1 in sputum (*n* = 10) or deep cough swab (*n* = 1) to 0 (V2 and V3) in deep cough swabs concurrent with the absence of any detectable S. aureus DNA in all samples at V2 and V3. For H. influenzae (*n* = 11), median bacterial load decreased from 0.43 to 0.02 (V2) and 0.003 (V3). In six patients, H. influenzae DNA was newly detected at V2 or V3 after a negative pre-HEMT examination, with a median load of 0.06.

### Evolution of biodiversity during the treatment with ELX/TEZ//IVA.

The analysis of the abundance of the CF pathogens in the patients’ airways was unfortunately biased in most cases by the divergent source, i.e., sputum at V1 and cough swabs at V2 and V3. Hence, to eliminate this bias, which prevented a valid statistical evaluation, we next assessed the longitudinal development of biodiversity in paired deep cough swab samples (Fig. 4). While a significant decline in bacterial load and increase in Pielou’s evenness were apparent from pre-HEMT to V3 (Fig. 4A and B), Shannon and Simpson diversity indices remained unaffected by ELX/TEZ/IVA (Fig. 4C and D). In terms of beta-diversity, a principal coordinate analysis of Euclidean distance matrices revealed similar airway microbial community profiles in CF patients prior and under ELX/TEZ/IVA therapy (Fig. 4E). However, samples obtained pre-HEMT exhibited significantly higher distances to their group centroid in a multivariate space than samples obtained at V2 and V3, suggesting that individual microbial community signatures pre-HEMT evolved to a more common signature after treatment commencement with ELX/TEZ/IVA (Fig. 4F).

### Prediction of treatment time point with random forest classification.

**FIG 5:**
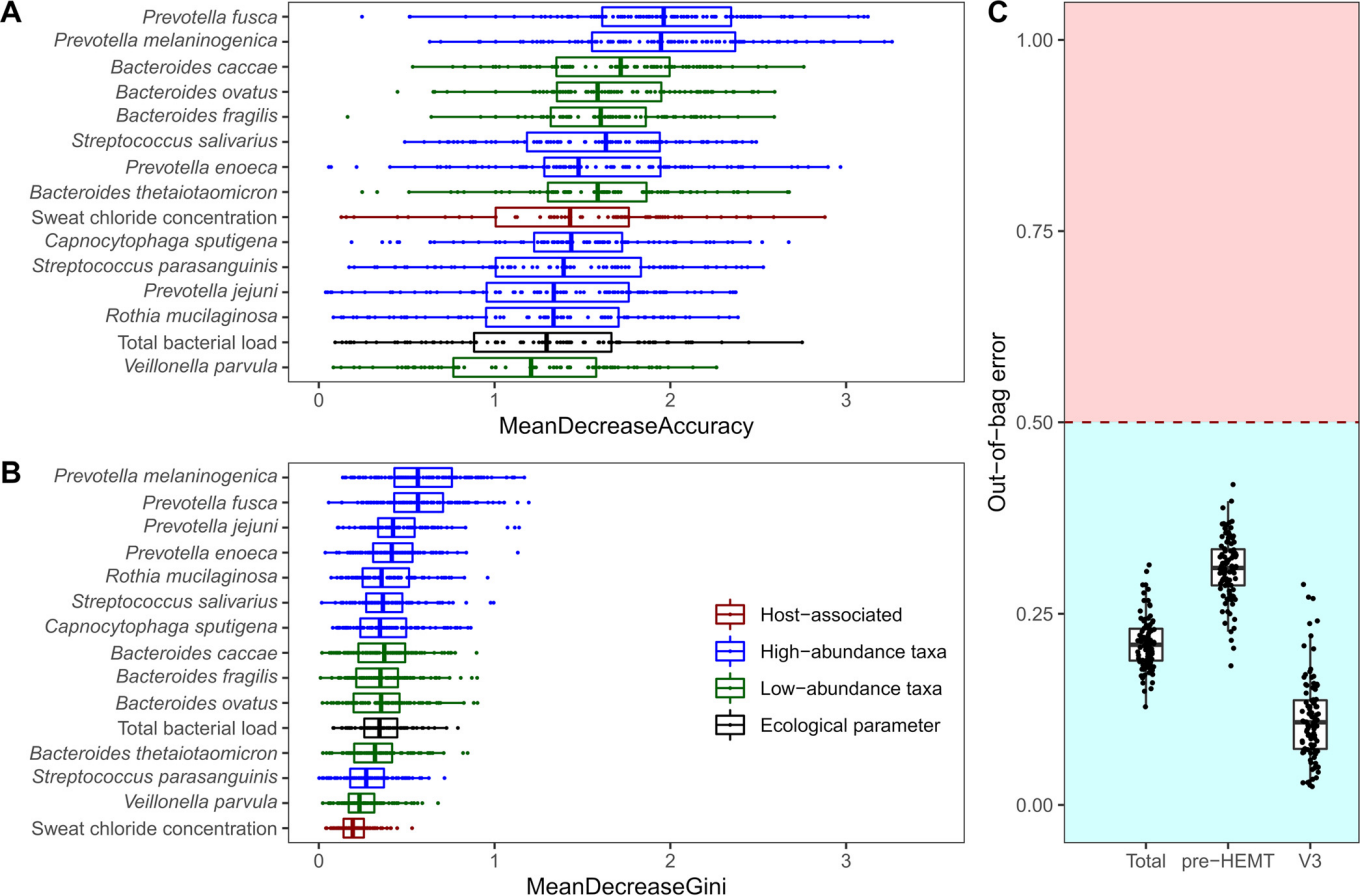
Extracting the nonrandom airway microbial metagenome and clinical features distinguishing pre-HEMT CF cough swabs (*n* = 13) from 13 randomly selected cough swabs after 50 weeks of HEMT with random forest bootstrapping aggregation. (A) Representation of the classification outcome based on the mean decrease accuracy. (B) Representation of the classification outcome based on the mean decrease Gini. (C) Overview of OOB estimates of error for random forest classifications, which were repeated 100 times with different seeds set for the classification and Boruta feature selection. The mean OOB estimate of error was 0.21 (standard deviation = 0.03) with mean class errors for pre-HEMT and V3 samples of 0.31 (standard deviation = 0.04) and 0.11 (standard deviation = 0.06), respectively. Note: input microbial metagenome variables included absolute abundances per taxa, Shannon and Simpson diversity indices, Pielou’s evenness indices, and species number. Clinical input features were age, gender, mutation type (F/F versus F/MF), chronic colonization with P. aeruginosa (culture-based diagnostics), previous modulator therapy (naϊve, LUM/IVA, TEZ/IVA), BMI, sweat chloride concentration, FEV, MEF_25_, and Sermet score (78).

A random forest bootstrapping aggregation of paired cough swabs was performed with the objective of extracting the key airway metagenome variables and clinical parameters distinguishing pre-HEMT, V2, or V3 samples. When pre-HEMT and 14 weeks of treatment with ELX/TEZ/IVA were compared, the CFTR biomarkers sweat chloride concentration and NPD Sermet score predicted the time point of sampling based on the mean decrease accuracy and Gini (Fig. S2). At the 1-year time point of treatment, the random forest classifier confirmed the significant effect of ELX/TEZ/IVA on the reduction of total bacterial load and the relative increase of the abundance of airway commensals (Fig. 5).

### Correlations between metagenome variables and clinical parameters.

**FIG 6:**
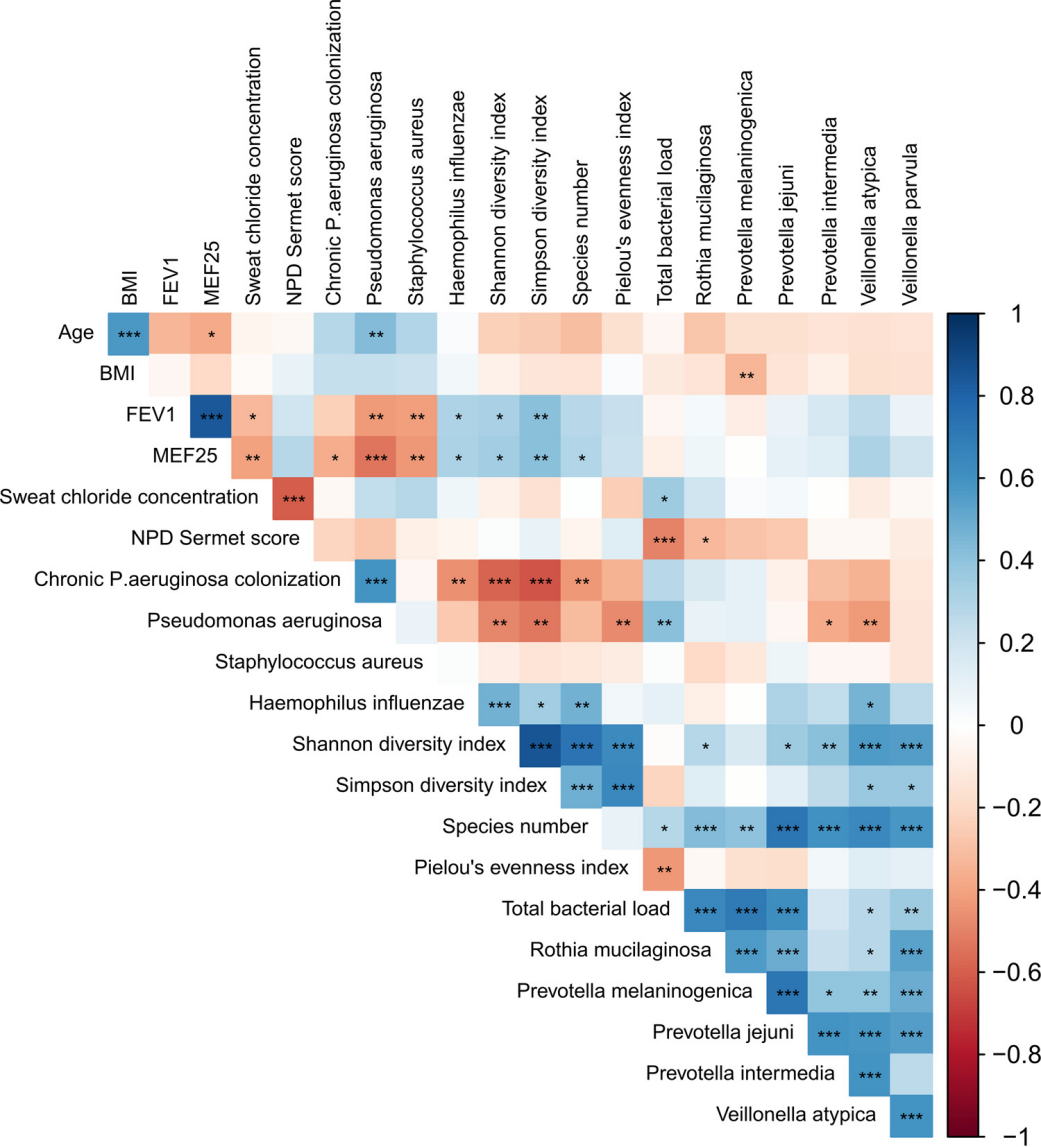
Spearman’s rank-order correlations of clinical and microbial metagenome parameters. Spearman's Rank-order correlations were obtained between patient characteristics (age, BMI), pulmonary function (FEV_1_ and MEF_25_), quantitative CFTR biomarkers (sweat chloride concentration and NPD Sermet score), culture-dependent colonization, and culture-independent abundance of common CF pathogens (chronic P. aeruginosa colonization, P. aeruginosa, S. aureus, and H. influenzae), metagenome and alpha-diversity parameters, including Shannon diversity index, Simpson diversity index, Species number, Pielou’s evenness index, bacterial load, and abundance of *Rothia* spp., *Prevotella* spp., and *Veillonella* spp. pre-HEMT and after 15 weeks of ELX/TEZ/IVA therapy (*, *P < *0.05; **, *P < *0.01; ***, *P < *0.001). *P*-values were adjusted for multiple testing by Benjamini-Hochberg correction.

To address the association of metagenome variables and clinical parameters, Spearman's Rank-order correlations were obtained between patient characteristics, including pulmonary function and quantitative CFTR biomarkers and metagenome parameters pre-HEMT and after 15 weeks of ELX/TEZ/IVA therapy (V2). Regarding our patient characteristics, small airway dysfunction, as determined by MEF_25_ in spirometry, correlated negatively with P. aeruginosa and S. aureus abundance and positively with H. influenzae abundance, Shannon diversity, Simpson diversity, and species number (Fig. 6). In terms of quantitative CFTR biomarkers, sweat chloride concentration correlated with S. aureus abundance and total bacterial load, whereas NPD Sermet score correlated negatively with total bacterial load, and *Rothia* spp. Abundance of P. aeruginosa correlated positively with age and bacterial load and negatively with MEF_25_, Shannon and Simpson diversity, Pielou’s evenness, and abundance of *Prevotella* and *Veillonella* spp. (Fig. 6).

### Effect of ELX/TEZ/IVA therapy on species cooccurrence networks.

**FIG 7:**
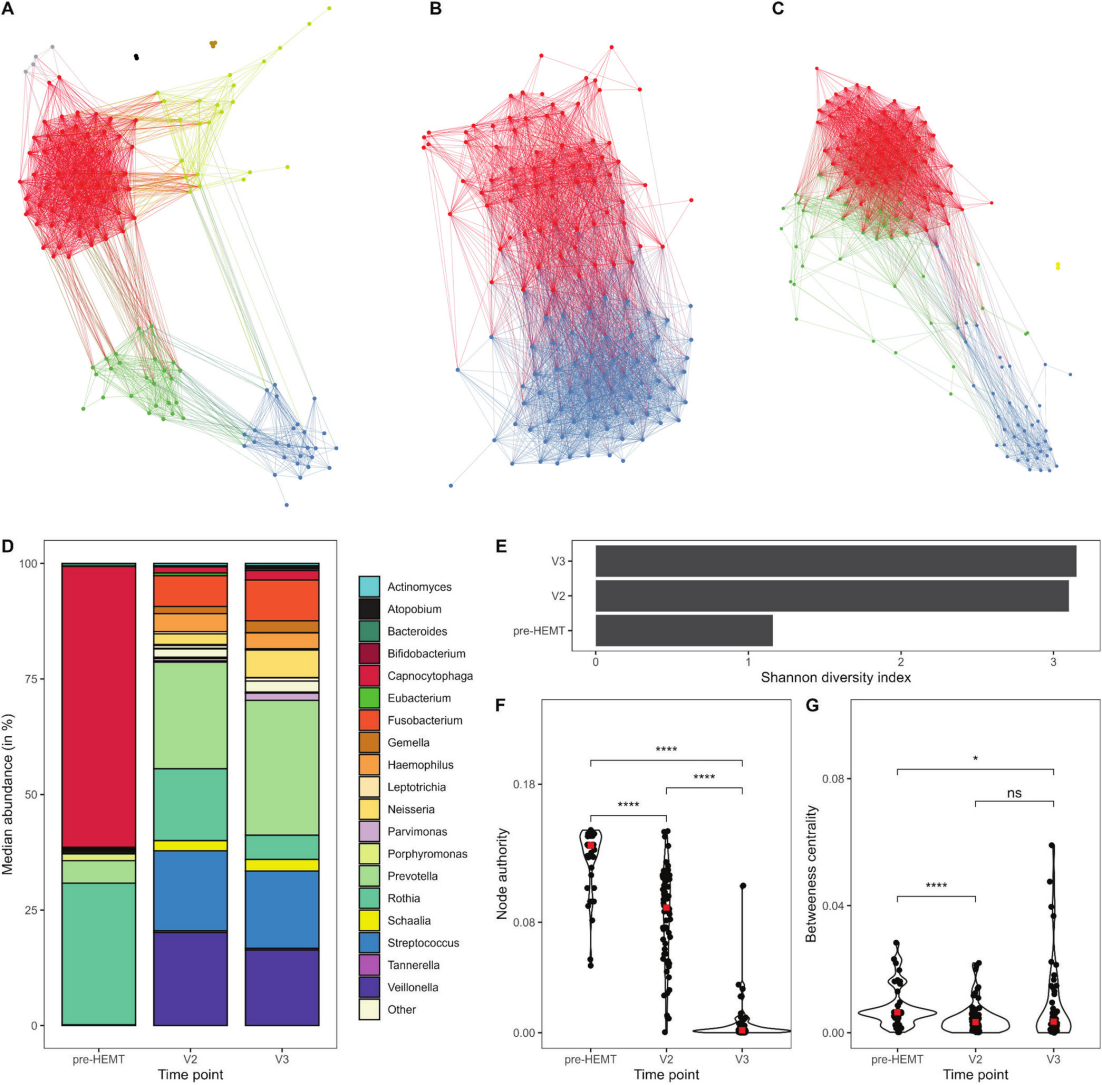
Evolution of species cooccurrence networks obtained from CF cough swabs before HEMT (*n* = 13) and after 14 weeks (V2, *n* = 27) and 50 weeks (V3, *n* = 24) of HEMT. (A) Visualization of the species cooccurrence network pre-HEMT. The seven observed modularity classes are depicted by red (40.7%), blue (18.6%), green (17.9%), yellow (16.4%), gray (2.9%), brown (2.1%), and black (1.4%) colors based on decreasing network contribution. (B) Visualization of the species cooccurrence network at the V2 study visit. The two observed modularity classes are depicted by red (51.8%) and blue (48.2%) colors based on decreasing network contributions. (C) Representation of the species cooccurrence network at the V3 study visit. The four modularity classes are depicted by red (43.6%), blue (28.6%), green (26.4%), and yellow (1.4%) colors based on decreasing network contributions. (D) Stacked bar-plot representation of the median species abundance (in %) in the largest network component at pre-HEMT, V2, and V3 (red color, Fig. 4A to C). (E) Visualization of changes in species diversity based on the Shannon diversity index of the largest network component pre-HEMT, V2, and V3. (F) Statistical comparison of Kleinberg’s Authority scores between the largest network modules pre-HEMT, V2, and V3. (Epsilon-squared effect size = 0.71; CI = 0.62–0.79). The Authority score of a node in a network represents the sum of all edges that point to this particular node (81). (G) Statistical comparison of Betweenness centrality scores between the largest network modulate pre-HEMT, V2, and V3 network structures (Epsilon-squared effect size = 0.1; CI = 0.04–0.2). The Betweenness centrality of a node is equal to the number of shortest paths between any two nodes in the graph passing through this node (72). Note: input arguments for running the ForceAtlas2 algorithm were the following: inertia = 0.1; repulsion = 2,000; attraction = 10.0; maximum displacement = 10.0; autostabilization = TRUE; gravity = 30.0; speed = 1.0. *, *P < *0.05; ****, *P < *0.0001.

Directed species cooccurrence network analyses of samples obtained pre-HEMT, V2, or V3 were conducted to identify the relevant ecological associations and structural community signatures before and after treatment commencement. By definition, cooccurrence networks represent the collective interconnection of taxa based on their paired presence (or absence) within a specified habitat. This information is deduced from the correlation of abundance patterns of aligned metagenome sequence data (72). Network properties are accessible to statistical testing, i.e., in our case, the impact of the intervention with ELX/TEZ/IVA on the cooccurrence network in the CF airways was assessed by changes in the Authority score and Betweenness centrality (see legend of Fig. 7).

At pre-HEMT, V2, and V3, global network structures were observed with seven, two, and four modularity classes, respectively (Fig. 7A to C, Table S5). Importantly, while the species cooccurrence network at V2 consisted of a single connected component (Fig. 7B), network fragmentation was apparent at pre-HEMT (Fig. 7A) and V3 (Fig. 7C). The largest network component at V1 was dominated by *Capnocytophaga* and *Rothia* spp. (Fig. 7D), in contrast to an increasingly diverse microbial community composition of the largest network components observed at V2 and V3 (Fig. 7E). Both Authority scores and Betweenness centrality decreased significantly from pre-HEMT to V2 and V3 (Fig. 7F and G). However, the reduction effect in Betweenness centrality was stronger from pre-HEMT to V2 than from pre-HEMT to V3 (Fig. 7G) indicating that the risk of network fragmentation decreased initially during treatment but reemerged after 50 weeks.

## DISCUSSION

This 1-year postapproval study examined the effect of combination therapy with the CFTR modulators ELX/TEZ/IVA on the composition of the CF airway metagenome. Compared to pre-HEMT, the total bacterial load decreased, the individual species were more evenly distributed in the community, and within the whole study cohort, the individual microbial metagenomes became more similar in their composition. In other words, we observed a conversion from a broad spectrum of divergent metagenomes ranging from monocultures to diverse communities of commensals toward microbial communities that are dominated by *Rothia*, Streptococcus, *Veillonella*, and *Prevotella* spp., the typical inhabitants of healthy airways (68, 69). An increase of evenness of the bacterial community during HEMT has already been reported for the sputum microbiome of CF adults who on average were 16 years older than our study participants (27).

As expected and consistent with current knowledge, the diversity and the abundance of commensals (3, 32, 40), measures of the CF defect in sweat duct and respiratory epithelium (62), as well as spirometry as an indicator of lung function (44, 47) correlated within their functional categories of metagenome, CFTR activity, and lung function, respectively. However, our analyses identified a few significant correlations between functional categories, namely, the influence of microbial diversity, S. aureus, and P. aeruginosa on lung function, more specifically, small airway dysfunction, as assessed by MEF_25_. The major improvement of lung function during triple modulator therapy was associated with a drastic reduction of the load with the pathogens S. aureus and P. aeruginosa in the whole cohort. In contrast to the experience gained with ivacaftor monotherapy in patients with a gating mutation, neither persistence nor regrowth of these two lead CF pathogens was observed in the cough swab metagenomes by the end of the 1-year study period (57–59). Considering the fact that our study participants represent the whole spectrum of very mild to severe CF lung disease, we consider it encouraging that ELX/TEZ/IVA combination therapy can convert the two major CF pathogens into rare members of the CF airway microbial community. Longer observation periods will be necessary to resolve whether the partial reversion of the basic defect that is achieved with ELX/TEZ/IVA is sufficient in the long-run to render the CF lungs robust against the recolonization with the common opportunistic pathogens. We should remain cautious with long-term extrapolation, because the cooccurrence network was strongly stabilized intermittently after 3 months of triple therapy but 9 months later had disintegrated into fragments that now more resembled the network pre-HEMT (Fig. 7A).

According to random forest analysis, sweat chloride and NPD score were the two most decisive factors governing the composition of the airway metagenome of our study population. Both parameters reflect CFTR activity (44, 62). Hence, we would like to conclude that the initial change of the CF airway metagenomes during the first weeks of HEMT therapy was primarily shaped by the incremental gain-of-function of mutant CFTR. The normalization of CFTR-mediated chloride conductance of the respiratory epithelium in the NPD correlated with the reduction of bacterial load in the cough swabs. This result fits with the recent report on the outcome of ivacaftor therapy in CF patients with the p.Arg117His mutation that the individuals with the strongest normalization of sweat chloride were most potent in the eradication of S. aureus and P. aeruginosa from their lungs (59).

Our study has limitations, most obviously concerning the number of patients and its monocentricity, precluding many statistical analyses of subgroups and parameters that could be informative for the treatment effects of ELX/TEZ/IVA. Moreover, methodological aspects hamper our conclusions. While we were previously able to collect induced sputum from all pancreatic-insufficient people with CF from our center (3), we had to acknowledge that only a minority of study participants was able to produce sputum even after sputum induction pre-HEMT and only one individual could mobilize secretions after initiation of ELX/TEZ/IVA. Hence, our metagenome analysis is biased by contamination with the oropharyngeal flora and a reduced sensitivity in the retrieval of microbes from the conducting airways occurred after initiation of ELX/TEZ/IVA. Based on our experience with the sampling of infants (40), deep cough swabs were accepted as the best compromise.

In summary, our postapproval study demonstrates that combination therapy with ELX/TEZ/IVA is successful in reducing the load with S. aureus and P. aeruginosa and converting the airway microbiota into a community of commensals 3 and 12 months after initiation of therapy. However, the microbial network remains vulnerable to fragmentation, with unfavorable trends after 12 months. Long-term studies will have to assess whether the impressive gain of CFTR function (62) results in sustainable changes in the airway microbial metagenome toward decreased dysbiosis, as a key determinant of disease progression in CF lung disease.

## MATERIALS AND METHODS

### Study design, participants, and clinical examinations.

Our prospective observational postapproval study (ClinicalTrials.gov Identifier: NCT04732910) was approved by the ethics committee of the Hannover Medical School (8922_BO_S_2020). We obtained written informed consent from all patients or their parents or legal guardians included in the study. Detailed inclusion and exclusion criteria are provided in the supplemental material. Airway metagenome samples (deep cough swabs and/or sputum samples) were collected pre-HEMT, 14 ± 5 weeks, and 50 ± 14 weeks after initiation of therapy with ELX/TEZ/IVA (Fig. 1). Sweat chloride concentration and nasal transepithelial potential difference (NPD) were assessed pre-HEMT and after 14 ± 5 weeks. Spirometry and anthropometry were monitored at all study visits. Details are provided in the supplement.

### Airway metagenome sample collection.

Deep cough swabs were collected after obligate coughs using sterile cotton swabs and placed in DNA LoBind Tubes (Eppendorf; no. 30120094) (40). The swabs were trimmed with sterile scissors and immediately stored at −80°C until further processing. Sputum was collected according to the CFFT Therapeutics Development Network standard operating procedure 530.00 (70) for sputum induction after inhalation of 3% NaCl in sterile tubes (Sarstedt; no. 62.547.004) and immediately stored at −80°C until further processing.

### Measures for the prevention of contamination and quality assurance.

To reduce DNA contamination, equipment was cleaned with 2% sodium hypochlorite solution (wt/vol) (40). Sample processing was performed in the UV-PCR workstation under aseptic conditions using disposable lab coats, sterile gloves, and mouth and hair protection as previously described (40). Negative controls were stored, processed, and sequenced in parallel with the patient samples to ensure continuous quality control of the experiments (Fig. S3).

### DNA extraction and fragmentation.

DNA was extracted from cough swabs as previously described (39). Frozen sputum was dissolved 1:5 in 4°C-cold PBS containing 2% (vol/vol) β-mercaptoethanol. Samples were incubated on a rocker (frequency 50/min) for 1.5 h in an ice bath and vortexed every 30 min. After centrifugation (3,800 × *g*, 10°C, 10 min), human cells were lysed in 5 mL 4°C-cooled bidest H_2_O and incubated for 15 min in an ice bath on the rocker. After centrifugation, samples were incubated with 180 units of DNase (Qiagen; no. 79256) for 60 min in a hybridization oven (Hybrid 2000, Saur, Tübingen) at 37°C and washed 3× with SE buffer (75 mM NaCl, 25 mM EDTA, pH 7.5). Bacterial cells were lysed in hard-to-lyse buffer (20 mM Tris/HCl, 2 mM EDTA, 1% [vol/vol] Triton X-100, pH 8.0) with 4 mg lysozyme for 30 min in the Hybrid 2000 at 37°C. Samples were heated to 56°C and incubated overnight with 0.6 mg proteinase K in the Hybrid 2000 at 56°C. DNA was extracted using the Macherey-Nagel NucleoSpin Tissue kit (740952.250). DNA concentration was measured with the Qubit dsDNA HS assay kit (Q32851; Invitrogen). For fragmentation, 75 ng DNA was set in 130 μL 0.1× TE (12090015; Invitrogen). Three-hundred-base pair fragments were generated with Covaris (Program 2, Table S5). Cleaning was performed using Ampure XP beads (A63881) as described previously (40).

### Library preparation and DNA sequencing.

Sequencing spike-ins (Sequins) as internal DNA reference standards (2%, wt/wt) were added to the sample DNA (71). Fragment libraries were prepared with NEBNext Ultra II DNA Library Prep kit for Illumina (E7645) and NEBNext unique dual index primer pairs (E64405) and a total of eight PCR cycles. The Illumina NovaSeq platform was used for sequencing (NovaSeq 6000 SP reagent kit v1.5, 300 cycles, paired-end reads; Illumina; 20028400).

### Taxonomic classification and normalization.

A whole metagenomic sequencing alignment pipeline was applied (73) for the taxonomic classification with default adjustments based on paired-end data. For each sample, raspir (version 1.0.2) (41) was run and filtered out microbial species with nonuniform read distributions toward the reference genome. To obtain absolute microbial abundance estimations, raw reads were normalized to both genome length and library size before the relative abundance of each species was divided by the fraction of sequins-associated reads in each sample (71).

### Statistical analysis.

Nonparametric Mann-Whitney U tests (effect size *r*) and the Kruskal-Wallis rank tests (epsilon-squared effect size) were applied for comparing two or more than two groups, respectively.

Microbial biodiversity of the CF airway habitat at pre-HEMT, V2, and V3 was assessed in absolute and relative microbial abundances, species number (S), Shannon and Simpson diversity indices, and Pielou’s evenness. Beta-diversity was evaluated by a principal coordinate analysis of Euclidean-distance matrices (74) obtained from log_10_-scaled absolute microbial abundance counts. Multivariate dispersion for samples at pre-HEMT, V2, and V3 was measured by calculating the distance of group members to the group centroid in a multivariate space.

Spearman’s Rank-order correlations were assessed as a nonparametric ranked-based pairwise correlation test with a significance level of 0.05 (*). Significance tests with *P* values were calculated for each pair of input features, and *P* values were adjusted for multiple comparisons using the Benjamini-Hochberg correction.

A random forest analysis based on bootstrapping aggregations was applied (41) to identify the key determinants distinguishing pre-HEMT samples from V2 and V3 samples (75, 76). The classification performance was validated with the out-of-bag (OOB) estimate of error rate, class errors and the Boruta algorithm (77). The Boruta algorithm generates shadow attributes by randomly shuffling the original input variables and iteratively comparing random with the corresponding original variables, so that nonrandom features of importance for the classification were distinguishable from random feature assignments (42). Random forest and Boruta wrapper application runs were repeated 100 times with different seeds set for the classification and for the feature selection procedure to avoid a selection-based bias (42).

For the ecological network analysis, the best practice guidelines for species cooccurrence network construction were followed (72). Spearman’s rank correlation matrices were generated from absolute microbial abundance counts per time point and all significant positive and negative correlations were extracted (Spearman’s *P* value ≤ 0.01, correlation coefficient greater than or equal to +0.2 or less than or equal to −0.2). The open-source software Gephi (78) was utilized for a directed network analysis with the continuous graph layout algorithm ForceAtlas2 (79). The Louvain method was applied to measure the division strength of networks into subcommunities in terms of modularity scores (80). Authority scores of nodes in the networks were calculated with an iterative hyperlink-induced topic search algorithm (81).

All data analysis was performed in R (82).

### Data availability.

Microbial sequencing data are stored in the European Nucleotide Archive (PRJEB51171). Coding scripts are available from https://github.com/mmpust/kaftrio-cf-airway-metagenome.
