# Supplementary material for: Impact of Elexacaftor/Tezacaftor/Ivacaftor Therapy on the Cystic Fibrosis Airway Microbial Metagenome
Source: Microbiol Spectr. 2022 Sep 26;10(5):e01454-22. doi: 10.1128/spectrum.01454-22 (PMC9602284; doi:10.1128/spectrum.01454-22)
Supplement: Supplemental file 1 — Download spectrum.01454-22-s0001.pdf, PDF file, 2.0 MB [file spectrum.01454-22-s0001.pdf]

# Impact of elexacaftor-tezacaftor-ivacaftor therapy on the cystic fibrosis airway microbial metagenome

Pallenberg, Sophia T.[1,2]#, Pust, Marie-Madlen[1,2], Rosenboom, Ilona[1], Hansen, Gesine[1,2], Wiehlmann, Lutz[3], Dittrich, Anna-Maria[1,2]*, Tümmler, Burkhard[1,2]*

[1]Department for Pediatric Pneumology, Allergology and Neonatology, Hannover Medical School, Hannover, Germany

[2]German Center for Lung Research, Biomedical Research in Endstage and Obstructive Lung Disease (BREATH), Hannover Medical School, Hannover, Germany

[3]Research Core Unit Genomics, Hannover Medical School, Hannover, Germany

**Running Title:** Effect of ELX/TEZ/IVA on CF Airway Metagenome

## Supplementary Material

#Address correspondence to Sophia T Pallenberg, pallenberg.sophia@mh-hannover.de

*Anna-Maria Dittrich and Burkhard Tümmler contributed equally to this work.

**Table S1. Patient characteristics, clinical data and CFTR biomarkers** (normal values marked with *)

| Patient | Gender | Age at V1 | Genotype | *P. aeruginosa* colonization | Pre-Modulator | BMI [kg/m$^2$] | | | FEV$_1$ (% predicted) | | | MEF$_{25}$ (% predicted) | | | Sweat Chloride Concentration [mmol/l] | | NPD Sermet Score | |
|---|---|---|---|---|---|---|---|---|---|---|---|---|---|---|---|---|---|---|
| | | | | | | V1 | V2 | V3 | V1 | V2 | V3 | V1 | V2 | V3 | V1 | V2 | V1 | V2 |
| ***CF-01*** | f | 16.0 | F/MF | free | - | 21.9 | 22.5 | 23.0 | 93* | 106* | 106* | 80* | 103* | 108* | 106 | 20* | -0.70 | 1.08* |
| ***CF-02*** | f | 17.2 | F/MF | chronic | - | 25.0 | 25.7 | 23.9 | 71 | 83* | 60 | 23 | 46 | 23 | 109 | 101 | -0.97 | -0.61 |
| ***CF-03*** | f | 29.0 | F/MF | chronic | - | 18.7 | - | - | 66 | - | - | 28 | - | - | 100 | - | -1.07 | - |
| ***CF-04*** | f | 21.3 | F/MF | chronic | - | 19.2 | 19.2 | 19.6 | 53 | 82* | 78 | 13 | 71 | 35 | 103 | 14* | -0.75 | -0.24 |
| ***CF-05*** | f | 13.8 | F/MF | never | - | 20.0 | 19.6 | 20.5 | 129* | 147* | 143* | 92 | 159* | 133* | 104 | 54 | - | 0.10 |
| ***CF-06*** | f | 13.7 | F/MF | intermittend | - | 19.4 | 22.9 | 21.9 | 110* | 126* | 132* | 73 | 106* | 131* | 110 | 40 | -0.72 | 0.92* |
| ***CF-07*** | f | 12.5 | F/F | chronic | - | 15.6 | 17.1 | 18.2 | 53 | 89* | 69 | 38 | 106* | 41 | 95 | 10* | -1.20 | 0.68* |
| ***CF-08*** | f | 14.0 | F/MF | never | - | 17.4 | 18.6 | 18.0 | 85* | 94* | 103* | 99* | 74 | 182* | 104 | 25* | -0.92 | 0.37* |
| ***CF-09*** | f | 13.6 | F/F | free | - | 19.8 | 18.7 | 18.8 | 107* | 112* | 114* | 92* | 99* | 114* | 104 | 27* | -1.67 | - |
| ***CF-10*** | f | 12.7 | F/MF | chronic | - | 17.9 | 18.3 | 19.1 | 101* | 117* | 118* | 62 | 91* | 105* | 88 | 41 | -2.00 | 0.24 |
| ***CF-11*** | f | 14.7 | F/MF | free | - | 18.5 | 18.8 | 17.8 | 104* | 115* | 111* | 66 | 111* | 103* | 113 | 65 | -1.79 | -0.72 |
| ***CF-12*** | f | 20.8 | F/MF | free | - | 16.6 | 18.1 | - | 46 | 76 | - | 15 | 46 | - | 92 | 38 | -1.20 | 0.18 |
| ***CF-13*** | f | 12.1 | F/MF | chronic | - | 14.2 | 16.5 | 18.4 | 63 | 83* | 78 | 32 | 40 | 31 | 98 | 70 | - | 0.34* |
| ***CF-14*** | f | 24.3 | F/F | chronic | LUM/IVA | 20.2 | 20.9 | 20.9 | 94* | 125* | 123* | 39 | 117* | 108* | 79 | 37 | -1.07 | -1.09 |
| ***CF-15*** | f | 15.4 | F/F | free | LUM/IVA | 18.0 | 20.1 | 20.8 | 69 | 82* | 90* | 32 | 35 | 43 | 82 | 67 | -0.70 | 0.04 |
| ***CF-16*** | f | 15.5 | F/MF | free | - | 18.0 | 17.9 | 18.6 | 113* | 126* | 124* | 87* | 116* | 103* | 99 | 44 | 0.03 | 0.42* |
| ***CF-17*** | f | 41.2 | F/F | chronic | LUM/IVA | 26.9 | 25.7 | 26.5 | 94* | 112* | 113* | 31 | 68 | 58 | 84 | 47 | -1.54 | - |
| ***CF-18*** | f | 14.2 | F/F | never | - | 15.2 | 16.7 | - | 74 | 103* | - | 35 | 78 | - | 89 | 25* | - | - |
| ***CF-19*** | m | 17.1 | F/MF | free | - | 20.2 | 20.3 | - | 84* | 115* | - | 36 | 106* | - | 108 | 90 | -0.79 | 0.45* |
| ***CF-20*** | m | 12.7 | F/MF | never | - | 15.1 | 16.8 | 17.7 | 89* | 118* | 117* | 53 | 128* | 118* | 115 | 50 | 0.01 | -0.01 |
| ***CF-21*** | m | 12.9 | F/MF | free | - | 21.4 | 23.2 | 22.8 | 72 | 86* | 88* | 28 | 28 | 52 | 108 | 36 | -0.21 | 0.29* |
| ***CF-22*** | m | 44.0 | F/MF | chronic | - | 28.1 | 29.5 | - | 82* | 87* | - | 22 | 22 | - | 102 | 53 | -0.69 | 0.03 |
| ***CF-23*** | m | 32.1 | F/F | free | - | 20.2 | 22.2 | 22.2 | 76 | 92* | 87* | 33 | 47 | 44 | 96 | 63 | -0.25 | 0.87* |
| ***CF-24*** | m | 31.4 | F/F | intermittend | LUM/IVA | 20.0 | 20.0 | 20.0 | 64 | 96* | 104* | 32 | 46 | 69 | 75 | 56 | -0.13 | 0.43* |
| ***CF-25*** | m | 44.8 | F/F | chronic | TEZ/IVA | 21.4 | 22.7 | 22.1 | 60 | 62 | 66 | 19 | 22 | 21 | 108 | 52 | -0.77 | 0.05 |
| ***CF-26*** | m | 13.9 | F/F | free | - | 15.8 | 16.1 | 16.1 | 95* | 116* | 113* | 71 | 211* | 187* | 98 | 30 | -1.23 | 0.15 |
| ***CF-27*** | m | 20.7 | F/MF | free | - | 20.1 | 21.7 | - | 80* | 98* | - | 49 | 68 | - | 86 | 53 | -1.33 | 0.09 |
| ***CF-28*** | m | 20.3 | F/MF | chronic | - | 22.2 | 22.6 | 21.9 | 101* | 106* | 108* | 86* | 98* | 97* | 82 | 31 | -1.36 | 0.86* |
| ***CF-29*** | m | 16.2 | F/F | never | LUM/IVA | 19.2 | 19.8 | - | 87* | 108* | - | 52 | 102* | - | 80 | 23* | 0.60* | -0.36 |
| ***CF-30*** | m | 22.1 | F/F | free | TEZ/IVA | 21.2 | 21.9 | 22.9 | 96* | 108* | 101* | 96* | 152* | 141* | 115 | 59 | -0.51 | 1.58* |
| ***CF-31*** | m | 19.2 | F/F | free | LUM/IVA | 23.8 | 23.8 | 22.9 | 99* | 99* | 102* | 95* | 91* | 91* | 104 | 64 | -0.62 | -0.21 |

## Table S2. Patient sample types and microbial parameters

| Patient | Sample | | | Species richness | | | Pielou‘s evenness | | | Shannon diversity | | | Simpson diversity | | | Total bacterial load | | | *P. aeruginosa* load | | | *S. aureus* load | | |
|---|---|---|---|---|---|---|---|---|---|---|---|---|---|---|---|---|---|---|---|---|---|---|---|---|
| | V1 | V2 | V3 | V1 | V2 | V3 | V1 | V2 | V3 | V1 | V2 | V3 | V1 | V2 | V3 | V1 | V2 | V3 | V1 | V2 | V3 | V1 | V2 | V3 |
| ***CF-01*** | Sputum | Swab | Swab | 16 | 108 | 127 | 0.30 | 0.58 | 0.50 | 0.82 | 2.74 | 2.43 | 0.35 | 0.86 | 0.74 | 17.99 | 35.11 | 3.03 | 0 | 0 | 0 | 2.16 | 0.05 | 0 |
| ***CF-02*** | - | Swab | Swab | - | 13 | 31 | - | 0.15 | 0.80 | - | 0.38 | 2.73 | - | 0.14 | 0.89 | - | 70.68 | 10.62 | - | 0.59 | 0.08 | 0 | 0 | 0 |
| ***CF-03*** | Sputum | - | - | 12 | - | - | 0.02 | - | - | 0.05 | - | - | 0.01 | - | - | 811 | - | - | 807 | - | - | 0 | - | - |
| ***CF-04*** | Sputum | Swab | Swab | 12 | 25 | 67 | 0.31 | 0.59 | 0.54 | 0.78 | 1.90 | 2.28 | 0.35 | 0.76 | 0.79 | 352 | 12.40 | 10.70 | 281 | 0.02 | 0 | 42.77 | 0 | 0 |
| ***CF-05*** | Swab | Swab | Swab | 54 | 114 | 34 | 0.81 | 0.67 | 0.85 | 3.25 | 3.18 | 3.00 | 0.95 | 0.94 | 0.93 | 50.34 | 135 | 3.35 | 0 | 0 | 0 | 0 | 0 | 0 |
| ***CF-06*** | Sputum | Sputum | Swab | 32 | 15 | 7 | 0.74 | 0.84 | 0.71 | 2.55 | 2.27 | 1.38 | 0.89 | 0.88 | 0.63 | 98.74 | 32.51 | 0.13 | 19.89 | 0 | 0 | 0 | 0 | 0 |
| ***CF-07*** | Swab | Swab | Swab | 87 | 42 | 27 | 0.44 | 0.58 | 0.87 | 1.96 | 2.16 | 2.86 | 0.75 | 0.78 | 0.92 | 12300 | 73.80 | 1.23 | 0.89 | 0 | 0 | 0 | 0 | 0 |
| ***CF-08*** | Swab | Swab | Swab | 80 | 71 | 47 | 0.63 | 0.64 | 0.77 | 2.76 | 2.72 | 2.98 | 0.89 | 0.87 | 0.90 | 29.14 | 39.64 | 1.02 | 0 | 0 | 0 | 0 | 0 | 0 |
| ***CF-09*** | Sputum | Swab | Swab | 60 | 26 | 35 | 0.79 | 0.74 | 0.86 | 3.24 | 2.42 | 3.07 | 0.95 | 0.87 | 0.93 | 60.44 | 19.61 | 26.92 | 0 | 0 | 0 | 0 | 0 | 0 |
| ***CF-10*** | Swab | Swab | Swab | 88 | 3 | 29 | 0.38 | 0.83 | 0.85 | 1.69 | 0.91 | 2.87 | 0.68 | 0.54 | 0.92 | 2283 | 0.39 | 6.18 | 0 | 0 | 0 | 0 | 0 | 0 |
| | Sputum | | | 31 | - | - | 0.46 | - | - | 1.57 | - | - | 0.69 | - | - | 76.02 | - | - | 0 | - | - | 0 | - | - |
| ***CF-11*** | Swab | Swab | Swab | 120 | 72 | 43 | 0.65 | 0.78 | 0.72 | 3.09 | 3.35 | 2.73 | 0.92 | 0.94 | 0.89 | 139 | 54.67 | 19.34 | 0 | 0 | 0 | 0 | 0 | 0 |
| ***CF-12*** | Sputum | Swab | - | 48 | 25 | - | 0.81 | 0.80 | - | 3.15 | 2.56 | - | 0.93 | 0.89 | - | 46.62 | 24.04 | - | 0 | 0 | - | 7.25 | 0 | - |
| ***CF-13*** | Swab | Swab | Swab | 16 | 10 | 11 | 0.75 | 0.72 | 0.79 | 2.07 | 1.66 | 1.89 | 0.82 | 0.72 | 0.80 | 198 | 6.45 | 0.21 | 0 | 0 | 0 | 0 | 0 | 0 |
| ***CF-14*** | Sputum | Swab | Swab | 23 | 57 | 18 | 0.29 | 0.65 | 0.90 | 0.92 | 2.62 | 2.61 | 0.45 | 0.87 | 0.91 | 198 | 93.48 | 0.07 | 42.37 | 0 | 0 | 0 | 0 | 0 |
| ***CF-15*** | - | Swab | Swab | - | 62 | 113 | - | 0.73 | 0.68 | - | 3.02 | 3.23 | - | 0.93 | 0.93 | - | 21.90 | 24.45 | - | 0 | 0 | - | 0 | 0 |
| ***CF-16*** | Swab | Swab | Swab | 119 | 0 | 24 | 0.67 | 0 | 0.91 | 3.20 | 0 | 2.90 | 0.93 | 1 | 0.93 | 55.38 | 0 | 1.71 | 0 | 0 | 0 | 0 | 0 | 0 |
| ***CF-17*** | Sputum | Swab | Swab | 53 | 26 | 35 | 0.66 | 0.91 | 0.85 | 2.62 | 2.96 | 3.03 | 0.87 | 0.94 | 0.93 | 54.53 | 4.27 | 8.76 | 16.00 | 0 | 0 | 0.04 | 0 | 0 |
| ***CF-18*** | Sputum | Swab | - | 13 | 124 | - | 0.63 | 0.58 | - | 1.61 | 2.81 | - | 0.71 | 0.90 | - | 19.69 | 50.26 | - | 0 | 0 | - | 8.22 | 0 | - |
| ***CF-19*** | Sputum | - | - | 34 | - | - | 0.88 | - | - | 3.11 | - | - | 0.94 | - | - | 7.06 | - | - | 0 | - | - | 0.03 | - | - |
| ***CF-20*** | Swab | Swab | Swab | 54 | 81 | 66 | 0.57 | 0.76 | 0.64 | 2.28 | 3.35 | 2.69 | 0.80 | 0.95 | 0.88 | 7.61 | 35.98 | 54.14 | 0 | 0 | 0 | 0 | 0 | 0 |
| ***CF-21*** | Sputum | Swab | Swab | 44 | 36 | 11 | 0.78 | 0.85 | 0.93 | 2.96 | 3.04 | 2.23 | 0.92 | 0.92 | 0.87 | 18.81 | 0.47 | 0.01 | 0 | 0 | 0 | 0.19 | 0 | 0 |
| ***CF-22*** | Sputum | Swab | - | 10 | 103 | - | 0.19 | 0.64 | - | 0.45 | 2.96 | - | 0.18 | 0.91 | - | 35.46 | 25.79 | - | 31.96 | 0.01 | - | 2.38 | 0 | - |
| ***CF-23*** | Sputum | Swab | Swab | 10 | 1 | 53 | 0.44 | - | 0.61 | 1.02 | 0 | 2.42 | 0.52 | 0 | 0.81 | 41.73 | 0.01 | 1.31 | 0 | 0 | 0 | 11.62 | 0 | 0 |
| ***CF-24*** | Sputum | Swab | Swab | 32 | 14 | 37 | 0.82 | 0.50 | 0.74 | 2.85 | 1.31 | 2.68 | 0.91 | 0.51 | 0.88 | 11.77 | 1.35 | 0.39 | 0 | 0 | 0 | 2.19 | 0 | 0 |
| ***CF-25*** | Sputum | Swab | Swab | 8 | 17 | 16 | 0.22 | 0.66 | 0.92 | 0.45 | 1.88 | 2.56 | 0.18 | 0.77 | 0.91 | 84.58 | 7.11 | 0.06 | 76.37 | 0.25 | 0 | 0 | 0 | 0 |
| ***CF-26*** | Swab | Swab | Swab | 137 | 44 | 27 | 0.62 | 0.82 | 0.84 | 3.04 | 3.11 | 2.77 | 0.92 | 0.93 | 0.91 | 32.94 | 7.11 | 2.62 | 0 | 0 | 0 | 0 | 0 | 0 |
| ***CF-27*** | Swab | - | - | 7 | - | - | 0.96 | - | - | 1.88 | - | - | 0.84 | - | - | 1.25 | - | - | 0 | - | - | 0 | - | - |
| ***CF-28*** | Swab | Swab | Swab | 19 | 53 | 20 | 0.74 | 0.61 | 0.71 | 2.19 | 2.44 | 2.11 | 0.82 | 0.83 | 0.83 | 11.16 | 22.91 | 2.21 | 0 | 0 | 0 | 0 | 0 | 0 |
| ***CF-29*** | Swab | Swab | - | 135 | 33 | - | 0.45 | 0.86 | - | 2.20 | 3.02 | - | 0.70 | 0.93 | - | 9.55 | 1.95 | - | 0 | 0 | - | 0.004 | 0 | - |
| ***CF-30*** | - | Swab | Swab | - | 33 | 33 | - | 0.83 | 0.82 | - | 2.89 | 2.86 | - | 0.92 | 0.91 | - | 2.46 | 0.92 | - | 0 | 0 | - | 0 | 0 |
| ***CF-31*** | Swab | Swab | Swab | 134 | 98 | 33 | 0.64 | 0.67 | 0.90 | 3.12 | 3.06 | 3.15 | 0.91 | 0.92 | 0.95 | 320 | 5.82 | 0.18 | 0 | 0 | 0 | 0 | 0 | 0 |

**Table S3. List of low abundant species,** summarized as 'others', contributing to 5% of total abundance of all species in all samples ordered by decreasing total abundance*

| | ***Species name*** | |
|---|---|---|
| *Schaalia odontolytica* | *Bacteroides thetaiotaomicron* | *Spirosoma aerolatum* |
| *Streptococcus gordonii* | *Bacteroides caccae* | *Flammeovirgaceae bacterium* |
| *Capnocytophaga haemolytica* | *Bacteroides caecimuris* | *Niastella koreensis* |
| *Prevotella intermedia* | *Lautropia mirabilis* | *Runella slithyformis* |
| *Gemella sanguinis* | *Odoribacter splanchnicus* | *Flavobacterium johnsoniae* |
| *Streptococcus pneumoniae* | *Bacteroides cellulosilyticus* | *Clostridium scatologenes* |
| *Campylobacter concisus* | *Campylobacter curvus* | *Treponema denticola* |
| *Fusobacterium nucleatum* | *Anaerostipes hadrus* | *Sphingobacteriaceae bacterium* |
| *Barnesiella viscericola* | *Blautia hansenii* | *Sebaldella termitidis* |
| *Prevotella dentalis* | *Actinomyces oris* | *Agarilytica rhodophyticola* |
| *Streptococcus intermedius* | *Hymenobacter swuensis* | *Paludibacter propionicigenes* |
| *Streptococcus constellatus* | *Roseburia hominis* | *Spirosoma radiotolerans* |
| *Prevotella ruminicola* | *Actinomyces radicidentis* | *Filimonas lacunae* |
| *Haemophilus parahaemolyticus* | *Bacteroides ovatus* | *Neisseria meningitidis* |
| *Streptococcus anginosus* | *Bacteroides vulgatus* | *Minicystis rosea* |
| *Veillonella parvula* | *Neisseria bacilliformis* | *Paenibacillus borealis* |
| *Eubacterium sulci* | *Chryseobacterium gallinarum* | *Streptobacillus moniliformis* |
| *Tannerella forsythia* | *Actinomyces naeslundii* | *Paenibacillus mucilaginosus* |
| *Streptococcus himalayensis* | *Butyrivibrio proteoclasticus* | *Clostridium saccharobutylicum* |
| *Fusobacterium hwasookii* | *Campylobacter gracilis* | *Labilithrix luteola* |
| *Leptotrichia buccalis* | *Butyrivibrio hungatei* | *Clostridium beijerinckii* |
| *Pseudopropionibacterium propionicum* | *Bacteroides dorei* | *Moorea producens* |
| *Alistipes finegoldii* | *Lachnoclostridium phytofermentans* | *Clostridium saccharoperbutylacetonicum* |
| *Gemella haemolysans* | *Clostridium lentocellum* | *Emticicia oligotrophica* |
| *Streptococcus thermophilus* | *Draconibacterium orientale* | *Bernardetia litoralis* |
| *Actinomyces meyeri* | *Dyadobacter fermentans* | *Clostridium pasteurianum* |
| *Bacteroides salanitronis* | *Alkalitalea saponilacus* | *Actinomyces gaoshouyii* |
| *Parabacteroides distasonis* | *Chryseobacterium indologenes* | *Selenomonas ruminantium* |
| *Parvimonas micra* | *Pontibacter korlensis* | *Flavisolibacter tropicus* |
| *Neisseria cinerea* | *Clostridium argentinense* | *Treponema putidum* |
| *Neisseria mucosa* | *Marinifilaceae bacterium* | *Megasphaera elsdenii* |
| *Aggregatibacter aphrophilus* | *Spirosoma montaniterrae* | *Pelosinus fermentans* |
| *Bacteroides helcogenes* | *Porphyromonadaceae bacterium* | *Fusobacterium varium* |
| *Filifactor alocis* | *Abiotrophia defectiva* | *Pedobacter steynii* |
| *Bacteroides fragilis* | *Chitinophaga pinensis* | *Treponema pedis* |
| *Selenomonas sputigena* | *Haliscomenobacter hydrossis* | *Nostocales cyanobacterium* |
| *Gemella morbillorum* | *Pedobacter ginsengisoli* | |
| *Neisseria elongata* | *Clostridium cellulovorans* | |

*sorted in descending order first by columns and then rows

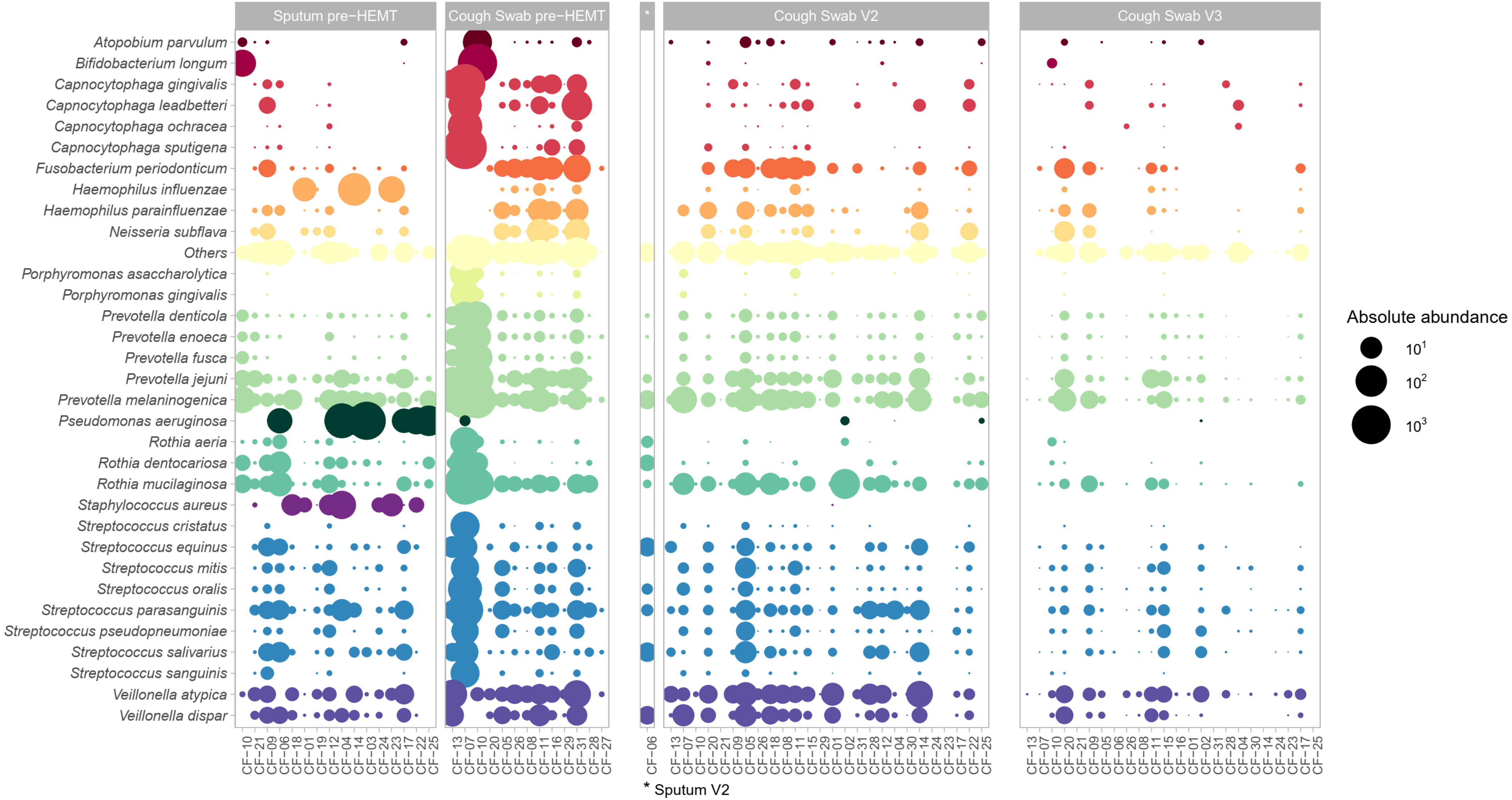


**Fig. S1. Overview of the absolute microbial abundances of core and rare species in CF airway metagenome samples pre-HEMT (V1) and after 14 weeks (V2) and 50 weeks (V3) of treatment with ELX/TEZ/IVA**. Note: Absolute abundances were calculated from sequins-normalized microbial count data (1). The species are sorted alphabetically. The colors represent taxonomic classification at genus level. Samples are ordered by sample type, time point (V1, V2 and V3) and age. Rare species are defined as all species contributing to a total abundance of <5% in all samples and are summarized as ‘others’. The * marks the sputum sample at V2.

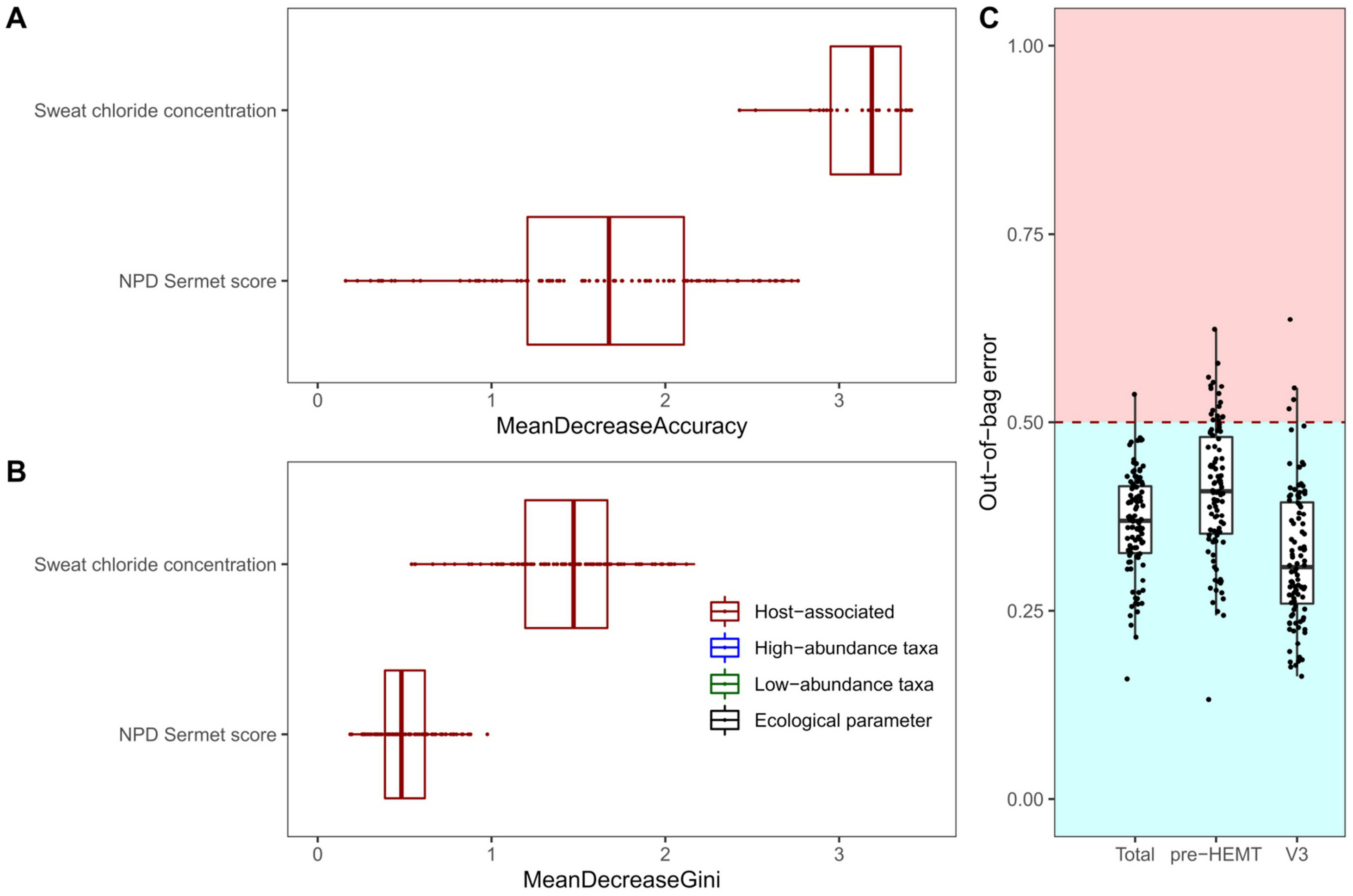


**Fig. S2. Extracting the non-random airway microbial metagenome and clinical features distinguishing pre-HEMT CF cough swabs (*n*=13) from 13 randomly selected cough swabs after 14 weeks of HEMT with random forest bootstrapping aggregation. (A)** Representation of the classification outcome based on the mean decrease accuracy. **(B)** Representation of the classification outcome based on the mean decrease Gini. **(C)** Overview of OOB estimates of error for random forest classifications, which were repeated 100 times with different seeds set for the classification and Boruta feature selection. The mean OOB estimate of error was 0.37 (standard deviation = 0.07) with mean class errors for pre-HEMT and V2 samples of 0.41 (standard deviation = 0.09), 0.32 (standard deviation = 0.09), respectively. Note: Input microbial metagenome variables included absolute abundances per taxa, Shannon and Simpson diversity indices, Pielou's evenness indices, and species number. Clinical input features were age, gender, mutation type (F/F vs. F/MF), chronic colonization with *P. aeruginosa* (culture-based diagnostics), previous modulator therapy (naïve, LUM/IVA, TEZ/IVA), BMI, sweat chloride concentration, $FEV_1$, $MEF_{25}$ and Sermet score (80).

**Table S4. Analysis of species co-occurrence networks**

| Network parameters* | V1 | V2 | V3 |
|---|---|---|---|
| Number of nodes (in total) | 140 | 143 | 140 |
| Number of edges (in total) | 4556 | 7324 | 5948 |
| Average Degree | 32.5 | 51.2 | 42.5 |
| Graph density | 0.23 | 0.36 | 0.31 |
| Connected Components | 3 | 1 | 2 |
| Average path length | 2.4 | 1.8 | 2.1 |
| Number of modularity classes | 7 | 2 | 4 |
| Contribution of the largest module | 40.7% | 51.8 % | 43.6 % |
| Average Degree (largest module) | 48.4 | 37.4 | 50.4 |
| Graph density (largest module) | 0.86 | 0.51 | 0.84 |

*ForceAtlas2 algorithm with the following criteria: Inertia = 0.1, repulsion strength = 2,000, attraction strength = 10, maximum displacement = 10, auto-stabilise function = True, Gravity = 30, Speed = 1.0

## Methods

### Inclusion Criteria:

- Patients with cystic fibrosis aged 12 years or older
- compound-heterozygous for p.Phe508del (F) and a minimal function (MF) mutation or homozygous for p.Phe508del
- no prior treatment with ELX/TEZ/IVA
- Patients were willing to maintain a stable drug regimen including ELX/TEZ/IVA for the duration of study participation

### Exclusion criteria:

- acute respiratory infection or pulmonary exacerbation at baseline
- history of transplantation

### Description of lung function and CFTR biomarker examinations

Sweat tests were performed according to the German national diagnostic guideline (2) and the guidelines of the Clinical and Laboratory Standards Institute (3). The skin on the forearm was stimulated by pilocarpine iontophoresis and sweat was collected with the Macroduct® system (Model 3700, Wescor, Logan UT, USA). Sweat chloride concentration was measured using a chloridometer (KWM 20 Chloridometer, Kreienbaum, Langenfeld, Germany) in a minimum volume of 30 µL.

NPD measurements were performed as previously described (4, 5, 6) and published recently for our study cohort (7). The Sermet Score was used to discriminate between normal (> 0.27) and reduced (< 0.27) CFTR function in the respiratory epithelium of the nose (4).

Spirometry was performed according to ATS/ERS standards and forced expiratory volume in one second ($FEV_1$) and mid-expiratory flow at 25% of vital capacity ($MEF_{25}$) was determined. Percent predicted results were based on equations of the global lung initiative (8).

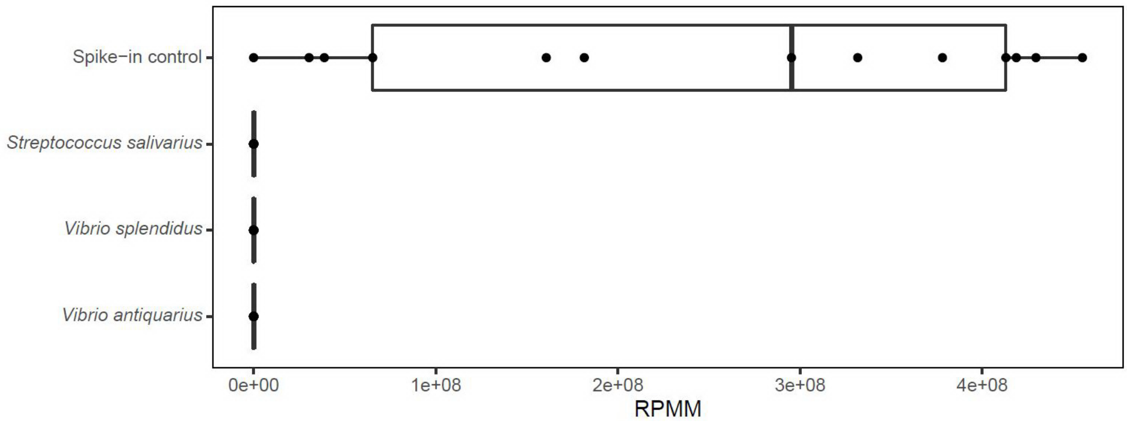


**Figure S3. The DNA background contamination of our laboratory environment.** Blank swabs (n = 10) and empty water controls (n = 3) were processed in parallel with patient samples. The microbial background contamination, which was here defined as microbial taxa detected in more than 10 % of negative samples after raspir filtering (09), was reduced to a minimum by implementing the proposed ultra-clean guidelines for shotgun metagenomic sequencing experiments (10). Next to the artificial spike-ins, only three microbes were detected, namely *Vibrio antiquarius, Vibrio splendidus, Streptococcus salivarius*. The lower and upper boundaries of the boxplot represent the first (25th percentile) and third (75th percentile) quartile. Whiskers extend from the box to the largest/smallest non-outlier data point (1.5 * IQR).

**Table S5. Programs for cell lysis (program 1) and DNA fragmentation (program 2) using the Covaris S220 Focused-ultrasonicator**

| | ***Program 1*** ***Cell lysis*** | ***Program 2*** ***DNA Fragmentation*** |
|---|---|---|
| Required run time | 3min 30 sec | 55 sec |
| Temperature | 5.0 – 8.0°C | 5.0 – 25.0°C |
| | Begin repeat (6x) | Begin |
| | 1. Treatment (5 sec) | 1. Treatment |
| Peak power | 200 | 140 |
| duty factor | 2.0 | 10.0 |
| cycles/burst | 100 | 200 |
| | 2. Treatment (30 sec) | End |
| Peak power | 275.0 | |
| duty factor | 5.0 | |
| cycles/burst | 100 | |
| | End repeat | |

**References:**


1. Hardwick SA, Chen WY, Wong T, Kanakamedala BS, Deveson IW, Ongley SE, Santini NS, Marcellin E, Smith MA, Nielsen LK, Lovelock CE, Neilan BA, Mercer TR. 2018. Synthetic microbe communities provide internal reference standards for metagenome sequencing and analysis. *Nat Commun*. 9:3096. doi: 10.1038/s41467-018-05555-0

2. Nährlich L, Stuhrmann-Spangenberg M, Barben J, Bargon J, Blankenstein O, Bremer W, Brunsmann F, Buchholz T, Ellemunter H, Fusch C, Gembruch U, Hammermann J, Jacobeit J, Jung A, Keim V, Loff S, Mayr S, Pfeiffer-Auler S, Rossi R, Sitter H, Stern M, Straßburg C, Derichs N. S2-Konsensus-Leitlinie „Diagnose der Mukoviszidose" (AWMF 026-023). 2013 6/30/2013 (cited 2015 9/29/2015).

3. Wayne PA. Sweat testing: sample collection and quantitative chloride analysis; approved guideline, 3rd ed. CLSI document C34-A3. Clinical and Laboratory Standards Institute 2009.

4. Sermet-Gaudelus I, Girodon E, Sands D, Stremmler N, Vavrova V, Deneuville E, Reix P, Bui S, Huet F, Lebourgeois M, Munck A, Iron A, Skalicka V, Bienvenu T, Roussel D, Lenoir G, Bellon G, Sarles J, Macek M, Roussey M, Fajac I, Edelman A. 2010. Clinical phenotype and genotype of children with borderline sweat test and abnormal nasal epithelial chloride transport. *Am J Respir Crit Care Med*.182:929-36.

5. Graeber SY, Dopfer C, Naehrlich L, Gyulumyan L, Scheuermann H, Hirtz S, Wege S, Mairbaurl H, Dorda M, Hyde R, Bagheri-Hanson A, Rueckes-Nilges C, Fischer S, Mall MA, Tümmler B. 2018. Effects of Lumacaftor-Ivacaftor Therapy on Cystic Fibrosis Transmembrane Conductance Regulator Function in Phe508del Homozygous Patients with Cystic Fibrosis. *Am J Respir Crit Care Med* 197:1433-1442.

6. Rowe SM, Clancy JP, Wilschanski M. 2011. Nasal potential difference measurements to assess CFTR ion channel activity. *Methods Mol Biol* 741: 69-86.

7. Graeber SY, Vitzthum C, Pallenberg ST, Naehrlich L, Stahl M, Rohrbach A, Drescher M, Minso R, Ringshausen FC, Rueckes-Nilges C, Klajda J, Berges J, Yu Y, Scheuermann H, Hirtz S, Sommerburg O, Dittrich AM, Tümmler B, Mall MA. 2022. Effects of Elexacaftor/Tezacaftor/Ivacaftor Therapy on CFTR Function in Patients with Cystic Fibrosis and One or Two *F508de*l Alleles. *Am J Respir Crit Care Med*. 205:540-549. doi: 10.1164/rccm.202110-2249OC.

8. Quanjer PH, Stanojevic S, Cole TJ, Baur X, Hall GL, Culver BH, Enright PL, Hankinson JL, Ip MS, Zheng J, Stocks J; ERS Global Lung Function Initiative. 2012. Multi-ethnic reference values for spirometry for the 3-95-yr age range: the global lung function 2012 equations. *Eur Respir J.* 40:1324-1343. doi:10.1183/09031936.00080312

9. Pust M-M, Tümmler B. 2021. Identification of core and rare species in metagenome samples based on shotgun metagenomic sequencing, Fourier transforms and spectral comparisons. *ISME Commun* 1:2.

10. Pust MM, Wiehlmann L, Davenport C, Rudolf I, Dittrich AM, Tümmler B. 2020. The human respiratory tract microbial community structures in healthy and cystic fibrosis infants. *NPJ Biofilms Microbiomes*. 6:61. doi: 10.1038/s41522-020-00171-7.
